# The senescence-like activity of BMS-470539 is associated with anti-fibrotic actions in models of dermal fibrosis

**DOI:** 10.1186/s13075-025-03635-3

**Published:** 2025-09-30

**Authors:** Camilla SA Davan-Wetton, Natalya Khodeneva, Christopher P. Denton, David J. Abraham, Mauro Perretti, Trinidad Montero-Melendez

**Affiliations:** 1 The William Harvey Research Institute, John Vane Science Centre Charterhouse Square Campus, Faculty of Medicine and Dentistry, Queen Mary University of London, London EC1M 6BQ, London, UK; 2 Department of Inflammation and Rare Diseases, UCL Centre for Rheumatology, Division of Medicine, University College London, London, UK; 3 Centre for Inflammation and Therapeutic Innovation, Queen Mary University of London, London, UK

**Keywords:** Melanocortin, Scleroderma, Fibroblast, Senescence, Resolution inflammation

## Abstract

**Background:**

Conditions like fibrosis, rheumatoid arthritis or cancer, once seen as distinct diseases, are now recognized to share strikingly common pathogenic mechanisms. The key to this convergence lies in the fibroblast, a pivotal driver of disease progression, tissue injury and chronicity. Despite this central pathogenic role and growing recognition as a therapeutic target, effective treatments targeting fibroblasts remain elusive, leaving a critical gap in disease intervention.

**Methods:**

Here we present a novel approach to target fibrosis using the melanocortin compound BMS-470539 to treat in vitro cultured human dermal fibroblasts obtained from healthy volunteers and systemic sclerosis patients and measuring various markers of senescence and fibroblast activation combining microscopy, DNA sequencing, cell migration and RNA sequencing and PCR techniques. We also used the in vivo bleomycin induced skin fibrosis in mice to determine pre-clinical efficacy of BMS-470539.

**Results:**

BMS-470539 induced a ‘senescence-like’ state in human dermal fibroblasts from systemic sclerosis patients, characterised by proliferation arrest, lack of pro-inflammatory secretome and inability to induce secondary senescence. This senescence-like activity (accompanied by β-galactosidase activity, lipofuscin accumulation and other markers) resulted in the downregulation of fibrosis markers including ⍺-smooth muscle actin, migration, CCL2 and genes related to TGFβ and fibroblast activation. In vivo, the compound reduced skin thickness on the bleomycin-induced skin fibrosis murine model when administered intraperitoneally, and importantly, this senescence-like activity did not cause signs of fibrosis when administered intradermally.

**Conclusions:**

Here, we introduce a novel strategy to disarm pathogenic fibroblasts in the context of skin fibrosis using a therapeutic pro-senescence-like approach using the unconventional melanocortin compound BMS-470539, to reset fibroblast behaviour and disrupt disease progression. This work also emphasizes the translational potential of how understanding shared pathogenic mechanisms across diseases may lead to new therapeutic opportunities to manage multiple diseases like arthritis and fibrosis.

**Supplementary Information:**

The online version contains supplementary material available at 10.1186/s13075-025-03635-3.

## Introduction

The traditional drug discovery pipeline relies on identifying molecular targets within the organ where the disease manifests. For instance, studying the liver in liver fibrosis, and the colon in colon cancer, assuming that pathology begins and ends at the affected site. But this organ-centric model is being fundamentally challenged. Emerging evidence reveals that diseases can originate far from their eventual site of damage, as seen in rheumatoid arthritis (RA), which may begin in the oral, intestinal, or lung mucosa before reaching the joints [1]. This shift in understanding demands a rethinking of how we define therapeutic targets.

Beyond redefining disease origins, precision medicine has transformed how we classify and treat conditions. Recognition of disease heterogeneity strongly influences the way we search for novel targets, consisting of searching for ‘*differences within a disease’* subtypes to tailor therapeutic approaches to the specific needs of each patient or patient subtype, a stratification strategy also being applied to RA [2]. But moving another step forward, new strategies that are transforming the way we approach diseases consist of searching for ‘*similarities across diseases’* to identify cellular or molecular mechanisms that may contribute to multiple diseases and hence be targeted in a similar way. Important lessons in this respect derive from the field of cancer research, where instead of grouping patients by affected organ (e.g. breast cancer or colon cancer), we now recognize that molecular signatures such as BRCA1/2 mutations or BRAF-V600E alterations, that transcend tissue boundaries, can provide more effective treatment strategies across cancers [3]. This molecular precision allows not only a more personalised approach but also to expand the translational applications of these new drugs into new indications. This cross-disease perspective is now reshaping other fields, revealing shared cellular and molecular mechanisms that underpin multiple conditions. The future of drug discovery lies not only in tailoring treatments to disease subtypes but in leveraging these commonalities to develop therapies with broader, more transformative impact. An example of this is the anti-interleukin 6 receptor antibody Tocilizumab, commonly used for rheumatoid arthritis, which recently gained approval for lung fibrosis [4].

These new advances in our understanding of pathological systems are transforming our approaches to disease, not only for cancer management but importantly for autoimmune diseases. Shared mechanisms, molecular and cellular, operate across these diseases and taking a holistic approach rather than a disease/organ focus to develop new treatments may lead to new therapeutic innovations. An important advancement towards this approach is exemplified by the Connect Immune Research Consortium [5], created in the United Kingdom to address shared mechanisms to autoimmune diseases, or the Accelerating Medicines Partnership^®^ Autoimmune and Immune-Mediated Diseases (AMP^®^ AIM) Program in the USA.

More specifically, the concept of the stromal cell as a therapeutic target is an example of how understanding shared cellular mechanisms of disease may help expand the translational opportunities of novel therapeutic interventions. The fibroblast has emerged in recent years as pathogenic driver and hence as a promising cellular target for a wide range of diseases, including RA, fibrosis or cancer, and specific therapeutic strategies are being developed [6–8]. In most cases, although not always, fibroblasts acquire an aggressive overactivated state that does not resolve, resulting in tissue damage. In RA, epigenetic imprinting [9] arrests these cells in a state of permanent activation, directly causing bone and cartilage damage, as well as continuous activation and recruitment of immune cells into the joints, a feedforward loop that leads to further damage and joint dysfunction. In fibrosis, activated fibroblasts (myofibroblasts) fail to enter apoptosis [10], getting locked in a state of continuous matrix deposition and crosslinking resulting in a gradual replacement of parenchymal cells with dysfunctional scar tissue. Furthermore, despite variations in triggering factors and manifestations, the underlying cellular and molecular events leading to fibrosis across different organs are fairly similar [11], suggesting the translational value of anti-fibrotic molecules may span various fibrotic conditions, irrespective of the affected organ.

This shared failure of fibroblasts to switch off offers a shared opportunity to develop innovative interventions that may be applied to a range of diseases. Strategies targeting the fibroblasts [6–8], not only will dampen the pathogenic actions directly exerted by them (i.e. cartilage destruction, excessive matrix deposition), but will also break the reinforcing crosstalk and reciprocal interactions with immune cells, which also contribute to the pathogenic circuits in the conditions described above.

We have previously reported the ability of the small molecule BMS-470539 to target synovial fibroblasts from RA patients [12]. This compound arrested RA fibroblasts proliferation with the concomitant expression of several markers of senescence, like senescence-associated β-galactosidase (SA-βGal) activity, accumulation of lipofuscin and presence of binucleated cells among others, along with a general reduction of its aggressive state. Moreover, we also observed these cells acquired a remodelling phenotype characterised by reduction in collagen production and upregulation of remodelling enzymes, prompting us to hypothesise that this molecule may have translational value for the treatment of fibrotic diseases.

Here, we propose BMS-470539 as a potential new therapeutic approach to fibrotic diseases and provide further characterization of the actions of this compound, originally described in 2003 as a selective melanocortin 1 receptor (MC_1_) agonist [13]. We also provide new insights into the unique features of this compound, compared to other melanocortin molecules, and how integrating and sharing knowledge across medical disciplines may have important translational value.

## Methods

### Primary human dermal fibroblasts

Human dermal fibroblasts were obtained from 4 mm skin punch biopsy specimens from the forearm, isolated as described by Shi-Wen et al. 2021 [14]. For systemic sclerosis (SSc), biopsies were collected from involved anterior forearm skin. Participating patients had all been diagnosed with SSc in line with the 2013 American College of Rheumatology/European League Against Rheumatism (ACR/EULAR) classification criteria for systemic sclerosis. Briefly, minced biopsies were incubated at 37 °C in 5% CO_2_ in Dulbecco’s Modified Eagle Medium (DMEM; Gibco) supplemented with 20% heat inactivated foetal bovine serum (FBS; Gibco). The biopsy cultures were assessed using brightfield microscopy until an outgrowth of fibroblasts could be seen. The derived fibroblasts were maintained in DMEM supplemented with 10% FBS, 2 mM glutamine, 100 U/ml penicillin and 100 µg/ml streptomycin (‘complete medium’) until ~ 80% confluence. Cells were sub-cultured at 1:3 split using 0.05% trypsin-0.02% EDTA solution for 5 min, before neutralization with complete DMEM. Cells were used between passages 3–15.

### Chemical compounds and treatments

The compounds used in this study were: BMS-470539 - CAS No. 2341796-82-3 (Tocris [for in vitro experiments] and Cayman Chemical [for in vivo experiments*]*), bleomycin (Cayman Chemical), alpha-melanocyte stimulating hormone ⍺MSH (Tocris), dersimelagon - CAS No. 1835256-48-8 (Merk), dimethyl sulfoxide (Merck), nintedanib (Tocris) and pirfenidone (Tocris). Bleomycin and nintedanib were reconstituted in DMSO, all other compounds were reconstituted in phosphate buffered saline (PBS, Gibco).

### Lysosomal detection by phase-contrast microscopy

Cells were treated with the compounds and over time periods as indicated in each figure legend. At the end of the period, cells were washed in PBS and images acquired using a Nikon Eclipse TE300 microscope and imaged with Image Pro Plus (Media Cybernetics, USA). The proportion of cells exhibiting a perinuclear ring were quantified.

### Lysosomal detection by immunofluorescence staining

LysoTracker™ (Thermo Fisher Scientific) was used according to the manufacturer’s instructions. The culture medium was removed from the cells, which were then washed twice in sterile PBS. The cells (unfixed) were then incubated in LysoTracker™ diluted to 75 nM in complete DMEM and incubated for 30 min at 37 °C in 5% CO_2_. The LysoTracker™ was removed and the cells washed once in PBS. Cells were counterstained with the nuclear dye Hoechst before visualising with a fluorescence microscope (EVOS FL Imaging System equipped with GFP Light Cube (green, EX470/22 - EM525/50), Thermo Fisher Scientific).

### Senescence-associated β-galactosidase (SA-βGal) staining

The Senescence Detection Kit (Abcam) was used according to manufacturer’s instructions. Cells were fixed with the provided fixative for 15 min. Staining solution containing 1 mg/ml 5-bromo-4-chloro-3-indolyl-β-D-galactopyranoside (X-Gal) was then added and cells incubated in sealed plates at 37 °C overnight (~ 16 h). Then, the staining solution was removed, cells washed with PBS and the development of blue colour indicative of SA-βGal activity visualized using a brightfield microscope (EVOS XL Core Imaging System, Thermo Fisher Scientific).

### Sudan black B (SBB) staining

A saturated SBB solution was prepared by dissolving 1.2 g of Sudan black B (Merck) in 80 ml of 70% ethanol [15]. Cells were fixed in 4% paraformaldehyde (PFA, Santa Cruz) for 15 min, before rinsing in 70% ethanol for 2 min. Cells were then incubated in saturated SBB solution for 8 min on an orbital shaker at 200 rpm. Cells were washed for 5 min in distilled water. In some cases, cells were counter-stained with nuclear fast red (Sigma). Lipofuscin accumulation was visualised using a brightfield microscope (EVOS XL Core Imaging System, Thermo Fisher Scientific) and a fluorescence microscope (EVOS FL Imaging System equipped with Cy5 Light Cube (far red, EX628/40 - EM685/40), Thermo Fisher Scientific).

### Immunofluorescence staining in cultured cells

Cells were fixed in 4% PFA (Santa Cruz) for 20 min at room temperature before the fixative was removed and the cells washed thrice for 10 min each in PBS. Cells were then incubated in 5% normal goat serum (Abcam) diluted in PBS for 1 h followed by incubation in primary antibody overnight at 4 °C. Cells were washed thrice for 10 min each in PBS before incubating in secondary antibody for 2 h at room temperature. The cells were washed twice in PBS for 10 min each, followed by a 10-minute incubation in a 1 µg/ml DAPI solution in PBS (Merck). The cells were then washed for a final 10 min in PBS before visualisation using the EVOS FL Imaging System (Thermo Fisher Scientific). The primary antibodies used in this study were: anti-MC_1_ (1:100, polyclonal, Alomone, Cat. No. AMR-020), anti-αSMA (1:200, clone 1A4, Dako) and anti-Myc tag Alexa Fluor 594 conjugate (1:100, clone 9B11, Cell Signalling Technology). The secondary antibodies used were: anti-rabbit and anti-mouse IgG Alexa Fluor 647. The fluorescence light cubes used were: Cy5 (far red, EX628/40 - EM685/40) was used to detect SBB-stained lipofuscin and Alexa Fluor 647; Texas Red (red, EX585/29 - EM628/32) was used to detect Alexa Fluor 594 conjugated antibodies; DAPI (blue, EX357/44 - EX447/60) was used to detect nuclei.

### Immunofluorescence staining in tissues

For the detection of MC_1_ in mouse skin, formalin-fixed paraffin-embedded tissue sections were deparaffinised and rehydrated before undergoing antigen retrieval by incubating slides in a sodium citrate buffer at 95 °C for 30 min. The sections were then blocked in 1% goat serum diluted in PBS for an hour, before overnight incubation in primary antibody (anti-MC_1_ at 1:100, Alomone, Cat. No. AMR-020) at 4 °C. The sections were washed thrice for 15 min per wash in PBS before incubation in secondary antibody (anti-rabbit IgG Alexa Fluor 647) for 2 h at room temperature. The sections were again washed thrice for 15 min per wash in PBS and then mounted in ProLong™ Diamond Antifade Mountant with DAPI (Thermo Fisher Scientific) and left to cure overnight at room temperature. Sections were imaged with a fluorescence microscope (EVOS FL Imaging System, Thermo Fisher Scientific) equipped with Cy5 Light Cube (far red, EX628/40 - EM685/40) to detect Alexa Fluor 647.

### Transfections of HEK293 cells

HEK293T (ATCC CRL-3216) cells were maintained in complete DMEM. Cells were transfected with human *MC1R*, *MC2R*, *MC3R*, *MC4R* or *MC5R* TrueORF cDNA clones (all from Origene) using Lipofectamine 2000 (Invitrogen) and OptiMEM media (Thermo Fisher Scientific) according to manufacturer’s instructions. 250 ng DNA was used to transfect 1.5 × 10^4^ cells per well of a 48 well plate. Cells were analysed 24 h after transfection by immunofluorescence.

### Alamar blue assay

Sterile 1X alamar blue reagent was prepared from a 10X stock solution (Thermo Fisher Scientific) in complete cell culture medium. Cell culture media was removed and replaced with 2 ml of the 1X alamar blue solution in a 6-well plate. Cells were incubated at 37 °C in 5% CO_2_. After four hours, 100 µl of the solution was transferred to a 96-well plate and the fluorescence measured in a NOVOstar fluorescence plate reader (BMG Labtech) with the excitation/emission (EX/EM) set to 560/590nm.

### Cell area quantification

Cells were visualised using a Nikon Eclipse TE300 microscope and imaged with Image Pro Plus (Media Cybernetics). For calculating cell size, images were taken at 20X magnifications and cell area quantified using the image processing package Fiji (National Institute of Health).

### Cell proliferation quantification

Cells were treated with the compounds as indicated in each figure legend. At the end of the treatment period, cells were washed in PBS and then detached using 0.05% Trypsin-EDTA (Thermo Fisher Scientific). Trypsin was deactivated with the addition of complete DMEM, and the cells in suspension were pelleted by centrifuging at 270 g for 5 min. The cell pellet was resuspended in 50 µl sterile PBS. 20 µl of the cell suspension was added to 80 µl Turk’s solution (0.01% Crystal Violet (Sigma) in 3% acetic acid) and 10 µl of this cell suspension loaded into a Neubauer chamber (Marienfeld Superior) to calculate the number of cells per ml.

### Enzyme linked immunosorbent assay (ELISA)

The following kits were used according to manufacturer’s instructions: CCL2, IL8 and IL6 uncoated ELISA kits (Thermo Fisher Scientific). Supernatants used to measure IL6 were diluted 1/10 before use, and supernatants used to measure IL8 and CCL2 were diluted 1/5.

### Gap closure assay

Cells were plated at a density of 2 × 10^4^ per well in a 6-well plate and grown in complete medium (DMEM supplemented with 10% FBS, 2 mM glutamine, 100 U/ml penicillin and 100 µg/ml streptomycin) for 24 h. Then, cells were treated with the compound every other day for 6 days, during which the cells formed a confluent monolayer. At the end of the treatment period, a scratch was then made using a P1000 tip. The media was removed, cells washed twice in PBS, and fresh media was added. Images were taken at 4X magnification using a Nikon Eclipse TE300 microscope at this point, and then again 24 h post-scratch. The gap size was calculated as a percentage of the original gap size.

### RNA extraction, real time PCR and primers

RNA from cultured cells was extracted using Direct-zol RNA MiniPrep. cDNA was synthesized (1 ug RNA) with SuperScript VILO MasterMix (Invitrogen). Real time-PCR was performed with Power SYBR Green PCR Master Mix (Applied Biosystems), on the ABI Prism 7900HT Sequence Detection System. Expression was calculated as 2^−ΔΔCt^ using *HPRT1* as the reference gene. The following QuantiTect primers (QIAGEN) used were: *HPRT1* (QT00059066), *CDKN1A* (QT00062090), *CDKN2A* (QT00089964). An annealing temperature of 60 °C was used for all primers.

### Whole genome RNA sequencing and functional analysis

RNA was extracted from cultured cells as described above, and samples sent to UCL Genomics (London, UK) to undergo bulk RNA sequencing. Samples were processed with Kapa mRNA HyperPrep kit (Roche) to create stranded libraries. Sequencing was performed on a NovaSeq 6000 (Illumina), using an S1 flow-cell and 100 cycle SBS sequencing kit. Fastq files were analysed using Partek Flow Genomic Analysis Software (release no. 10.0.23.0720). Reads were aligned to the human genome hg38 using STAR 2.7.8a. Quantification to transcriptome was conducted using assembly Homo sapiens (human) hg38, annotation model RefSeq Transcripts 93 (2020-02-03). Transcript quantification was conducted with TPM normalisation method. Differentially expressed genes were obtained by comparing BMS-470539 treated with vehicle control treated, using a *p* = 0.05 as cut-off, yielding a working list of 1896 differentially expressed genes (990 upregulated and 906 downregulated). The Database for Annotation, Visualization, and Integrated Discovery (DAVID) classification tool (v2024q2) was used to perform the functional analysis.

### Genomic DNA extraction

Genomic (g)DNA was extracted using QIAamp DNA Mini Kit (Qiagen) according to the manufacturer’s advised protocol. The gDNA was eluted in 150 µl of RNase/DNase-free water. Quantity (ng/µl) and quality of the collected gDNA was measured using a NanoDrop™ 2000 (Thermo Fisher Scientific).

### *MC1R* genotyping

The entire coding region of *MC1R* was amplified in two fragments, using the primers Forward: 5’-GCCCAGATGGAAGGAGGC-3’ and Reverse: 5’-CATGAGCACCAGCATAGCCA-3’ (fragment 1) and Forward: 5’-CTGCTTCCTGGGCGCCAT-3’ and Reverse: 5’-CAGAGATCATTTAGTCCATCCTC 3’ (fragment 2). PCR products were purified and subjected to Sanger sequencing at Eurofins Genomics service (Ebersberg, Germany). Sanger sequencing was performed using ABI 3730xl DNA Analyzer and basecaller KB v1.4.1.8. Electropherograms were analysed using *ab1 files and variants detected using MacVector with Assembler (v18.7.0) against the *Homo sapiens MC1R* reference sequence NC_000016.10. Classification of variants according to red hair colour (RHC) phenotype is based on classification reported on Pena-Vilabelda et al. [16].

### Animals

Animal studies using the bleomycin model of dermal fibrosis were approved and performed under the guidelines of the Ethical Committee for the Use of Animals, University College London, and Home Office regulations (Guidance on the Operation of ASPA 1986). All experimental protocols complied with the relevant ethical regulations and were conducted under the Project Licence (PPL) PBA1ECDE5, using C57BL/6J mice purchased from Charles River. Female mice were used to better reflect the distribution of scleroderma in the human population, with a female to male ratio around 4:1.

Animal studies using *Mc1r*^e/e^ mice (The Jackson Laboratory) were approved by and performed at Queen Mary University of London under the guidelines of the Local Ethics committee and the UK Home Office under licences PPL70/8264 and PPL70/7986. C57BL/6NCrl were purchased from Charles River.

### Bleomycin-induced skin fibrosis murine model

To induce skin fibrosis, mice were injected intradermally with 50 µl of either 1 mg/ml (equal to 1 unit) of bleomycin or vehicle control (saline) into a single 1 cm^2^ shaved area on the back every other day for 28 days. To assess the therapeutic effects of BMS-470539 in this model, mice who were receiving intradermal bleomycin were injected with 100 µl of BMS-470539 at 18 mg/kg or vehicle control (PBS), administered intraperitoneally, from day 14 every other day until day 28. To compare the effects of intradermal administration of bleomycin vs. BMS-470539, a group of mice were injected intradermally with 50 µl of 3.6 mg/ml BMS-470539 prepared in saline every other day for 28 days, i.e. to match the bleomycin administration protocol.

At day 28, the mice were sacrificed by cervical dislocation. 6 mm punch biopsies were collected from the fibrotic lesions. Livers, kidneys and spleens were also collected for additional histological analyses. Cardiac punctures were undertaken on freshly sacrificed mice to obtain whole blood samples.

### Histological analyses

Tissues for histological analyses were fixed in 4% neutral buffered formalin and embedded in paraffin. Tissues were kindly embedded and sectioned at 5 μm thickness at the Barts Cancer Institute Pathology Services. Sections were deparaffinised in Histoclear II followed by decreasing concentrations of ethanol (100 − 50%) and then rehydrated in distilled water. For H&E staining, the sections were incubated in haematoxylin solution (Sigma) for 5 min, rinsed in running tap water and then counterstained with Eosin Y solution (Sigma) for 3 min. Stained sections were dehydrated and then mounted in Entrellan™ rapid mounting medium (Sigma) using 24 × 24 mm coverslips (VWR) and left to cure overnight. For picro sirius red staining and Masson’s trichrome staining, picro sirius Red Stain Kit (Abcam) and Trichrome Stain kit (Abcam), respectively, were used in accordance with the manufacturer’s instructions. Stained slides were imaged using a NanoZoomer S210 Digital slide scanner (Hamamatsu).

### Analysis of murine whole blood samples

Blood was obtained from cardiac puncture and aliquots (30 µl) were analysed using a ProCyte Dx Haematology Analyzer (IDEXX) to quantify white blood cell, reticulocyte and red blood cell counts.

### Statistics

All statistical analysis was conducted on GraphPad Prism 10 (GraphPad Software). All statistical parameters and relevant statistical tests used for each experiment are described in each figure legend. Data are always expressed as mean ± standard error of the mean (SEM), with *p* < 0.05 considered to be statistically significant. For all experiments, an n number is provided, representing the number of biological replicates unless otherwise stated. Generally, t-test or one-way ANOVA were used when applicable depending on the number of groups to be compared, and multiple comparisons test applied when relevant, which are specified in each figure legend. Asterisks or hash symbols are also included to denote the statistical significance reached in each case.

## Results

### The melanocortin compound BMS-470539 displays a senescence-like activity on human dermal fibroblasts

**Fig. 1:**
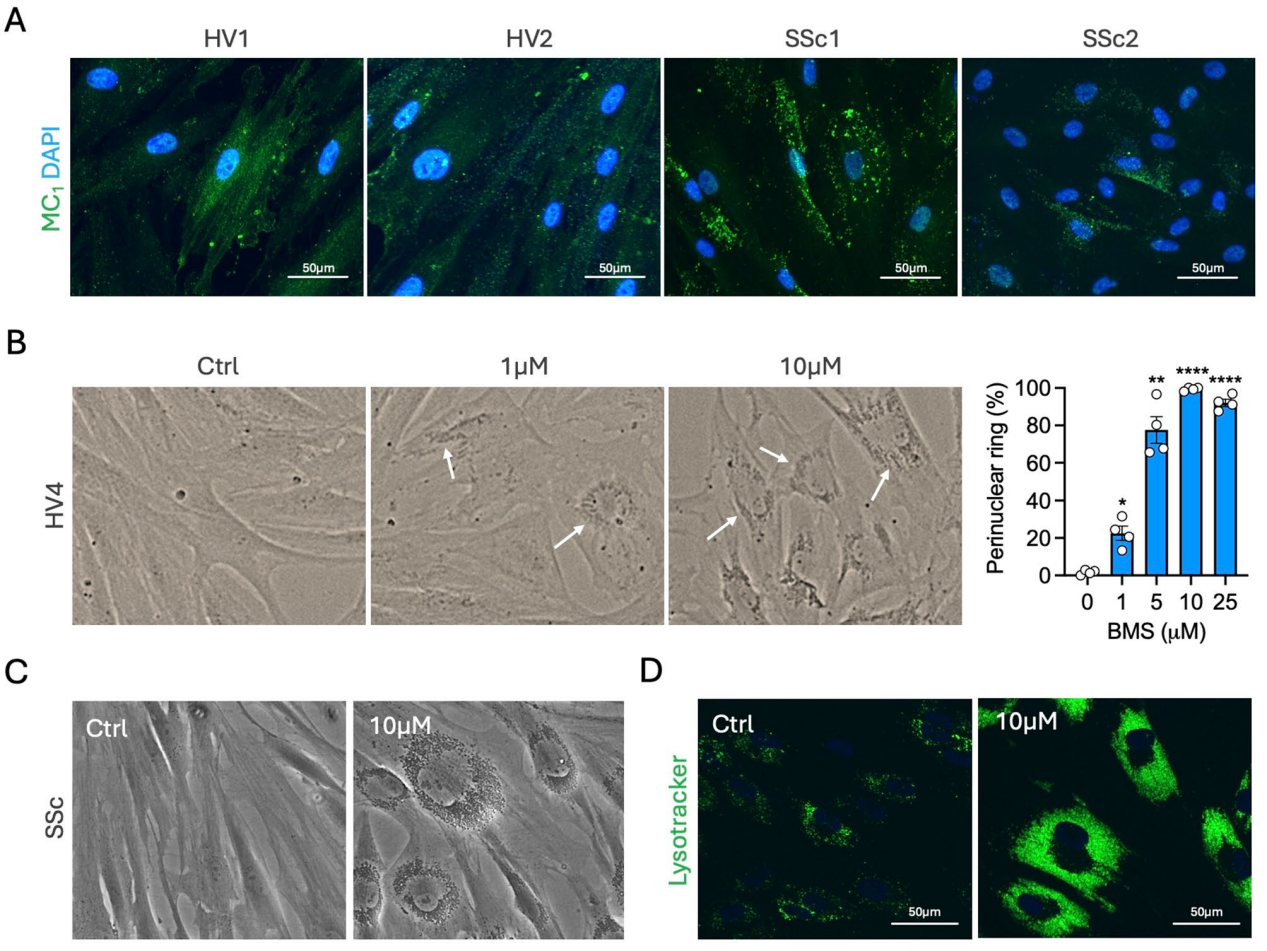
MC_1_ receptor expression on in vitro cultured dermal fibroblasts and response to MC_1_-selective agonist BMS-470539. Dermal fibroblasts obtained from healthy volunteers (HV) and systemic sclerosis patients (SSc) were used. (**A**) Expression of the melanocortin receptor type 1 (MC_1_) by immunofluorescence. (**B**) Healthy volunteers (HV) fibroblasts were treated with BMS-470539 every other day for 6 days at the indicated concentrations and visualised by light microscopy. The percentage of cells displaying a perinuclear lysosomal ring was quantified. **C**, **D**) SSc fibroblasts were treated with BMS-470539 every other day for 6 days at the indicated concentrations and visualised by light microscopy (**C**) and lysosomes stained with LysoTracker™ and visualised on a fluorescence microscope on the GFP (EX470/22—EM525/50) channel. Data represent mean ± SEM of *n* = 4 and was analysed by paired one-way ANOVA vs. control followed by Dunnett’s multiple comparisons test - *p < 0.05, **p < 0.01, ****p < 0.0001. Images were captured at 10X (light microscopy) or 40X (fluorescence microscopy) and scale bars represent 50 μm

**Fig. 2:**
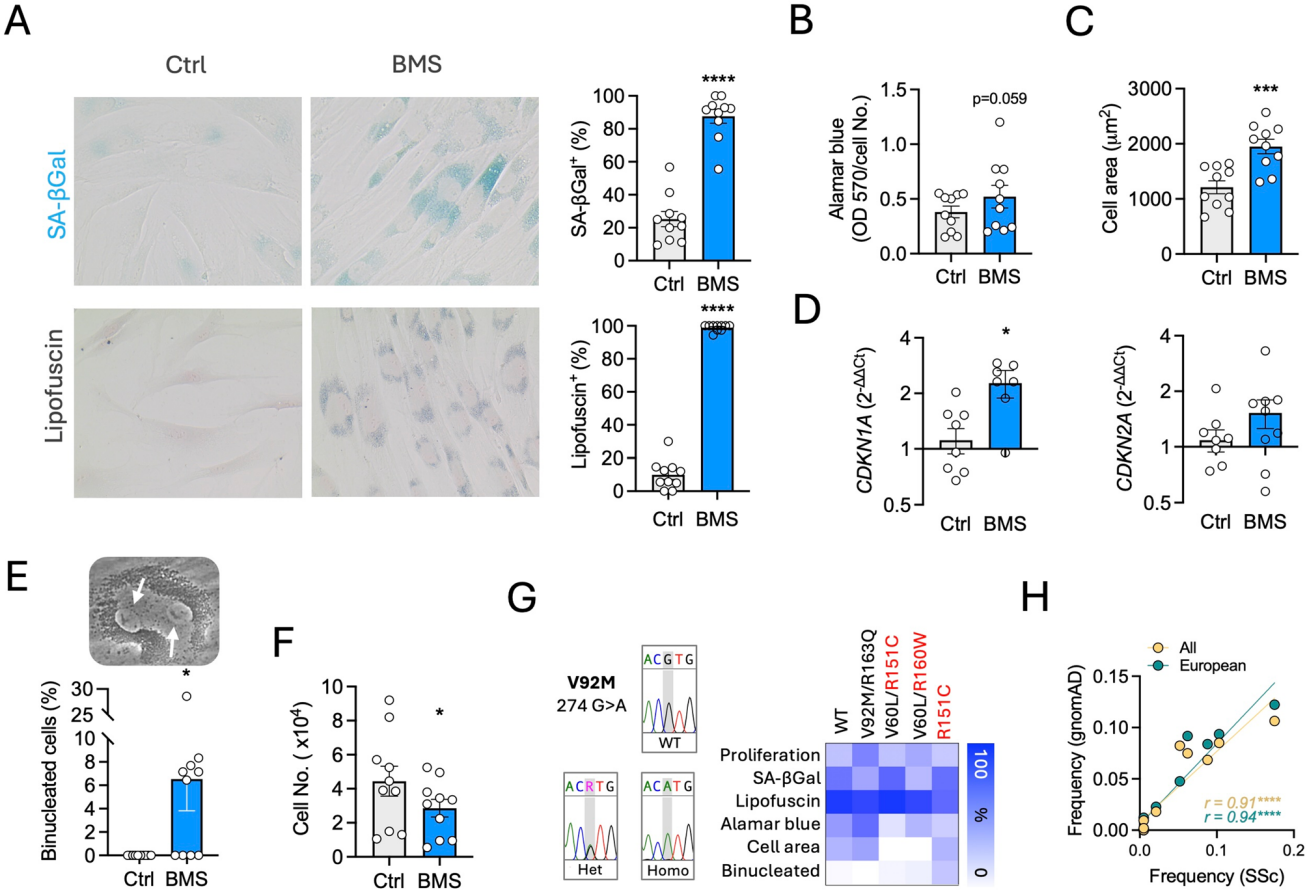
Senescence phenotypic markers are induced by BMS-470539 on in vitro cultured human dermal fibroblasts. Systemic sclerosis (SSc) dermal fibroblasts were treated with BMS-470539 every other day for 7 days at 10 µM. (**A**) Cells were stained for senescence associated β-galactosidase (SA-βGal) activity or lipofuscin using Sudan black B, and the proportion of cells positive for each marker quantified. (**B**) After the treatment period, cells were incubated in alamar blue solution for further 4 h and optical density measured at 570 nm. Data is normalised by cell number. (**C**) Cell size was estimated by measuring the area of each cell from light microscopy images using Fiji imaging processing tool. (**D**) Gene expression quantification by real time PCR of cyclin-dependent kinases 1 A (*CDKN1A*, or p21) and 2 A (*CDKN2A*, or p16). (**E**) The percentage of cells containing two nuclei was quantified. (**F**) Measurement of proliferative capacity by cell counting. (**G**) Representation of markers of senescence shown above based on the *MC1R* genotype. Data represent the percentage of change of BMS-470539 with respect to vehicle (Ctrl). An example of Sanger sequencing results for variant V92M is shown. (**H**) Analysis of the frequency of *MC1R* variants of SSc patients (*n* = 97) compared to variants frequency found in the general population and European (non-Finnish) populations according to data obtained from gnomAD database (v4.1.0). Data related to fibroblasts experiments represent mean ± SEM of *n* = 9–10 and was analysed by paired t-test - *p < 0.05, ***p < 0.001, ****p < 0.0001

BMS-470539 is a small molecule with selective agonistic activity at the melanocortin 1 receptor (MC_1_). Previously, we reported the induction of a senescence-like phenotype by this compound on human synovial fibroblasts [12], characterised by prominent perinuclear accumulation of lysosomes, senescence-associated β-galactosidase (SA-βGal) activity and reduced proliferation, among other senescence markers. Here, we tested this compound on human dermal fibroblasts obtained from both healthy volunteers (HV) and scleroderma patients (SSc) (see Supplementary Table 1 for donors and patients’ information). First, we confirmed the presence of MC_1_ receptor in these cells (Fig. 1A) using a selective antibody (full in-house validation provided in Supplementary Figs. 1,2). Addition of BMS-470539 to HV fibroblasts induced a concentration-dependent effect on the accumulation of lysosomes after a 6-day treatment period (Fig. 1B), reaching a maximum effect at 10 µM. The same effect was observed in SSc fibroblasts (Fig. 1C) and lysotracker staining confirmed the identity of these organelles as lysosomes (Fig. 1D). Further assessment of senescence markers on SSc cells treated with 10 µM BMS-470539 demonstrates increased in SA-βGal and lipofuscin accumulation (Fig. 2A), maintenance of metabolic activity (Fig. 2B), increase in cell size (Fig. 2C), upregulation of cyclin-dependent kinase inhibitors *CDKN1A and CDKN2A* (Fig. 2D), presence of binucleated cells (Fig. 2E) and reduced cell proliferation (Fig. 2F). The activity of BMS-470539 was not dependent on any particular genotype, at least related to the variants identified in this study (Fig. 2G, Supplementary Table 2), which is consistent with our previous findings in RA synovial fibroblasts. The genotyping of a cohort of SSc patients (*n* = 97) also revealed that the frequency of *MC1R* variants in SSc patients is highly similar to that of the general or European population (Fig. 2H, Supplementary Tables 3,4). However, neither the melanocortin pan-agonist ⍺MSH nor the MC_1_ selective molecule dersimelagon (also known as MT-7117) were able to induce this phenotype on dermal fibroblasts (Supplementary Fig. 3). Collectively, these data suggest that BMS-470539 induces a senescence-like phenotype in both healthy and diseased human dermal fibroblasts, which is unique to this melanocortin molecule.

### BMS-470539 reduces markers of fibrosis on human dermal fibroblasts

**Fig. 3:**
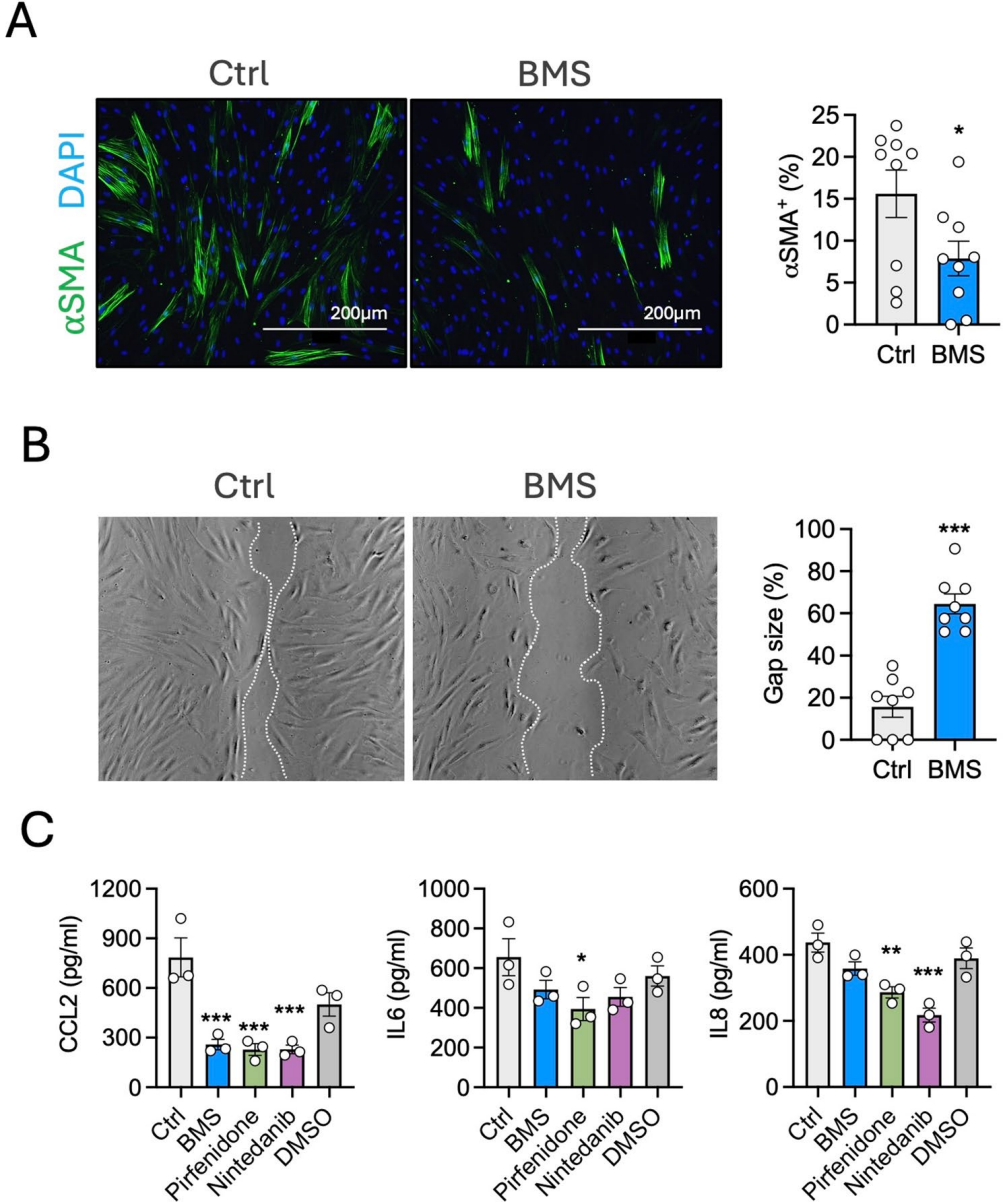
In vitro anti-fibrotic activity of BMS-470539 on cultured human dermal fibroblasts. (**A**) Systemic sclerosis (SSc) fibroblasts were treated with 10 µM BMS-470539, every other day for 6 days and αSMA expression analysed by immunofluorescence. (**B**) To address migratory activity, a scratch was created on cells previously treated with 10 µM BMS-470539 for 6 days and incubated for further 24 h in fresh media. Images were acquired on a light microscope and gap size quantified using Fiji imaging processing tool. (**C**) SSc fibroblasts were treated with 10 µM BMS-470539, 0.5 mM pirfenidone, 0.5 µM nintedanib or appropriate vehicle control (PBS or DMSO), every other day for 6 days. Cytokines were measured in supernatants by ELISA. Data (**A**, **B**) represent mean ± SEM of *n* = 8–9 and was analysed by paired t-test *p < 0.05, ***p < 0.001. Fluorescent images were captured at 10X and scale bars represent 200 μm. Data (**C**) represent mean ± SEM of *n* = 3 and was analysed by paired one-way ANOVA vs. control followed by Dunnett’s multiple comparisons test - *p < 0.05, **p < 0.01, ***p < 0.001

Having demonstrated the pro-senescence-like activity of BMS-470539 on HV and SSc dermal fibroblasts, we next investigated the potential of this compound in modulating functional responses of fibroblasts with relevance in fibrosis. Alpha-smooth muscle actin (⍺SMA) is one of the most important markers of myofibroblast differentiation and it is associated with fibroblasts contractile ability. In the SSc cohort of fibroblasts used in this study, approximately 15% of cells on average exhibited prominent ⍺SMA expression (Fig. 3A). This was reduced by almost half (7.9%) when cells were treated with BMS-470539. Similarly, the application of the compound significantly reduced cell ability to migrate, as assessed using the gap closure assay (Fig. 3B). The process of cellular senescence is typically associated with the release of a pro-inflammatory secretome (known as senescence-associated secretory phenotype, SASP). Here, we demonstrate that BMS-470539 does not promote this activity but rather leads to a decrease of inflammatory mediator release, particularly on CCL2, an effect that was similar to that obtained with known anti-fibrotic therapeutics like pirfenidone and nintedanib (Fig. 3C). In addition to propelling local inflammation, a typical SASP can also induce secondary senescence on surrounding cells. Here we confirm, using conditioned media obtained from vehicle treated and from BMS-470539 treated cells (Supplementary Fig. 4A), that media from BMS-470539 induced senescent cells do not induce further senescence on fresh cells (Supplementary Fig. 4B).

### Transcriptomic changes on fibroblasts treated with BMS-470539

**Fig. 4:**
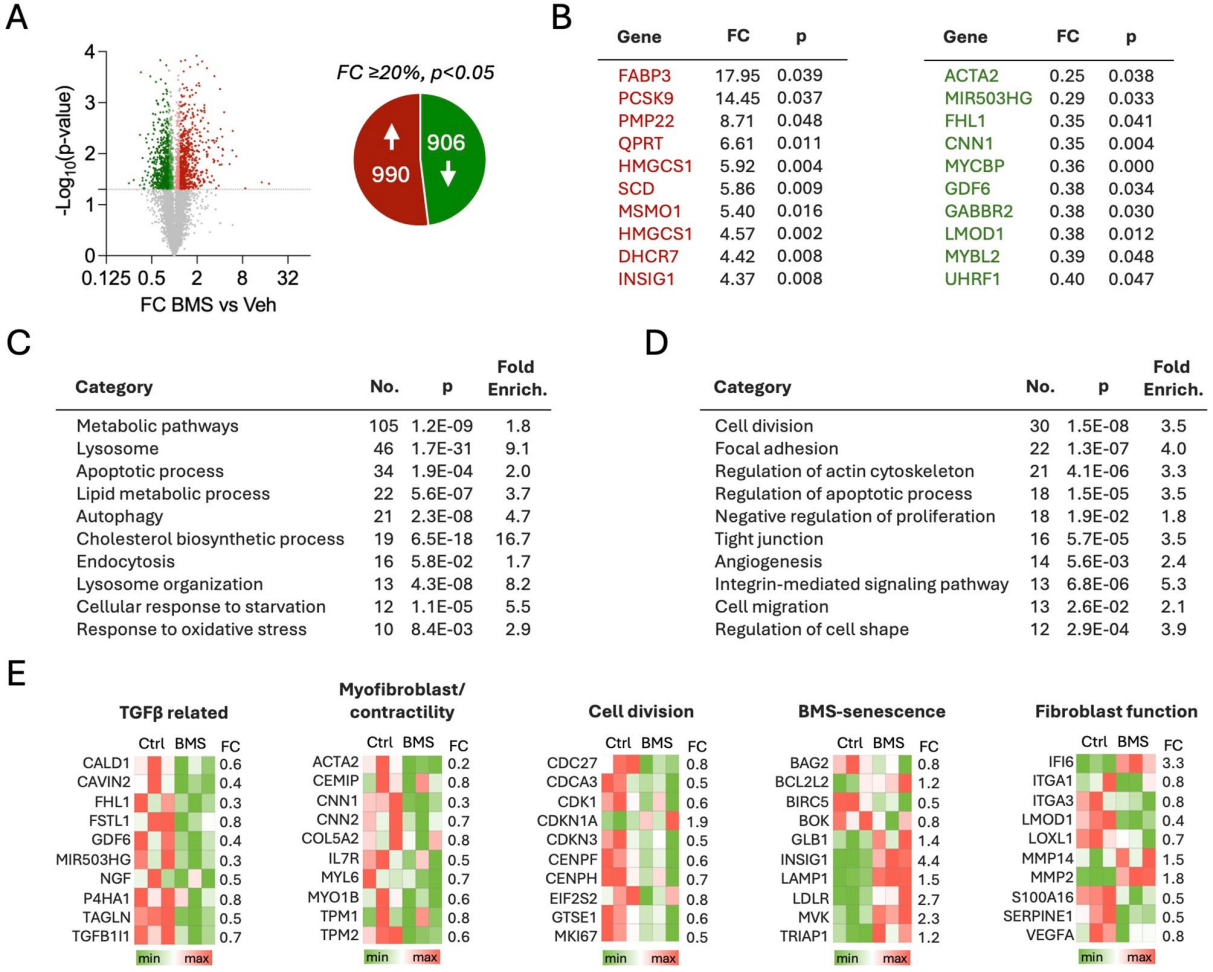
Transcriptomic analysis on cultured human dermal fibroblasts. **A**) **C**-**E**) Healthy volunteers (HV) fibroblasts (*n* = 3) were treated with 10 µM BMS-470539, every other day for 6 days. RNA was extracted and subjected to whole genome RNA sequencing. (**A**) A total of 1896 genes were found differentially expressed using a fold change (FC) of at least 20% change and *p* < 0.05 as cut-off criteria for selection. (**B**) The top 10 most upregulated and downregulated genes are shown. Functional analysis using the Database for Annotation, Visualization, and Integrated Discovery (DAVID) classification tool (v2024q2) is presented for up-regulated (**C**) and down-regulated genes (**D**). The number of genes (No.), *p*-value (p) and fold enrichment for each category are shown. Examples of genes are shown as heatmaps (**E**), and additional details provided in Supplementary Table 5. Data (**A**, **B**) represent mean ± SEM of *n* = 8–9 and was analysed by paired t-test *p < 0.05, ***p < 0.001. Fluorescent images were captured at 10X and scale bars represent 200 μm

To gain an unbiased and broader overview of cellular processes and functions affected by BMS-470539, a whole-genome transcriptomic study was performed to determine the transcriptomic effects of the drug. Fibroblasts were treated with 10 µM BMS-470539, every other day for 6 days, using vehicle (PBS) as controls. RNA was then extracted and subjected to RNA sequencing. After normalization and background filtering, a total of 1,896 individual transcripts were found to be differentially expressed (*p* < 0.05 and fold change greater than 20%, Fig. 4A) in BMS-470539 treated cells, compared to control vehicle treated cells. Remarkably, the most strongly down-regulated gene was *ACTA2*, the gene encoding for ⍺SMA, consistent with the protein expression data discussed above (Fig. 4B). A functional classification was performed using DAVID Functional Annotation tool. Among the up-regulated genes (Fig. 4C), cellular processes and pathways related to lysosomal accumulation, metabolism and cholesterol related pathways were highly enriched, in line with our previous findings on synovial fibroblasts [12]. Up-regulated expression was observed on genes related to lysosomal activity and cholesterol metabolism, like low density lipoprotein receptor (*LDLR*) or lysosomal associated membrane protein 1 (*LAMP1*); the gene encoding for SA-βGal (*GLB1*), metalloproteases (*MMP2*, *MMP14*), and anti-apoptotic BCL related genes (*BCL2L*, *BOX*) (Fig. 4C, E). On the other hand, genes implicated in processes like cell division, apoptosis, cell migration or integrin pathways were enriched in the downregulated subset of genes (Fig. 4D). Specific genes are presented in Fig. 4E and Supplementary Table 5. For example, we identified a consistent downregulation on genes directly associated with TGFβ activity, including caldesmon (*CALD1*) [17], caveolae associated protein 2 (*CAVIN2*) [18], transgelin (*TAGLN*) [19], and the long non-coding RNA *MIR503HG* [20], all involved in endothelial-to-mesenchymal transition. Downregulated expression was also observed for growth differentiation factor 6 (*GDF6*) -a TGFβ receptor ligand also known as BMP13-, or the gene *TGFB1I1* encoding for transforming growth factor beta 1 induced transcript 1. Other downregulated genes relevant to fibrosis include genes related to myofibroblast contractility like *ACTA2*, and others like calponins (*CNN1*, *CNN2*), myosins (*MYL6*, *MYO1B*), tropomyosins (*TPM1*, *TPM2*) or cell migration inducing hyaluronidase 1 (*CEMIP*). Multiple genes involved in cell division were also found down-regulated as well as genes related to fibroblast functions like integrins (*ITGA1*, *ITGA3*), which are implicated in cellular mechanotransduction; the calcium binding protein *S100A6*, with a known pathogenic role in fibrosis, the gene encoding for the plasminogen activator inhibitor-1 PAI-1 (*SERPINE1*), considered a myofibroblast marker, and the pro-angiogenic protein *VEGFA* among others. *LOXL1*, an enzyme involved in collagen cross-linking which contributes to tissue stiffness was also downregulated by BMS-470539.

### BMS-470539 attenuates bleomycin-induced skin fibrosis in mice

**Fig. 5:**
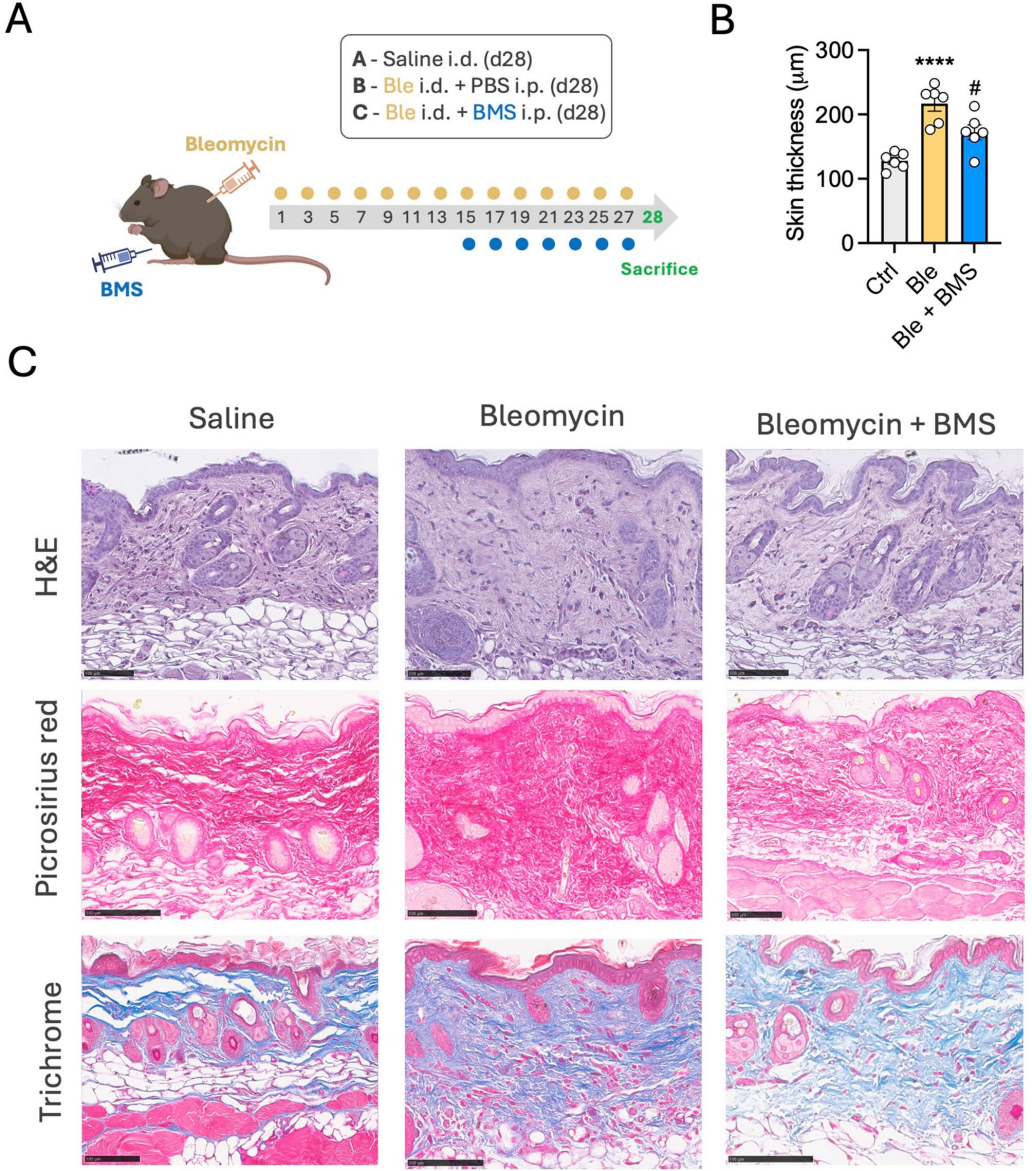
In vivo anti-fibrotic actions of BMS-470539 in the bleomycin-induced skin fibrosis murine model. (**A**) Diagram representing the experimental design used to test the therapeutic antifibrotic actions of BMS-470539. 1 mg/ml bleomycin was administered intradermally (i.d.) from day 1 to induce skin fibrosis while 18 mg/kg BMS-470539 was administered intraperitoneally from day 15. (**B**) The dermal thickness of the H&E-stained sections was quantified in NDP.view2 in vehicle (Ctrl), bleomycin i.d. (Ble) or bleomycin i.d. + BMS-470539 i.p. (Ble + BMS) treated mice (**C**) Representative images of H&E, picrosirius red and Masson’s trichrome stained tissue sections of skin from saline, bleomycin and bleomycin + BMS-470539 treated mice. Data represent the mean ± SEM of *n* = 6; one-way ANOVA, corrected with Tukey’s multiple comparisons test vs. Ctrl ( ****p < 0.0001) and vs. Ble (^#^*p* < 0.05). Images were captured using a Nanozoomer slide scanner and scale bars represent 100 μm

In order to establish the translational potential of the unique senescence-like activity of this molecule in the context of fibrotic diseases, we tested BMS-470539 in murine model of bleomycin-induced skin fibrosis. Briefly, 50 µl of 1 mg/ml bleomycin solution was injected intradermally every other day from day 1 to day 27, and mice sacrificed at day 28. BMS-470539 was administered intraperitoneally (i.p.) from day 15, once fibrosis had been established. Mice receiving PBS i.p. served as controls (Fig. 5A). An additional group of mice was sacrificed at day 14 to confirm establishment of fibrosis (Supplementary Fig. 5A). At day 14, the quantification of the dermal layer thickness on the H&E-stained sections demonstrate that the skin of the bleomycin treated mice was significantly thicker than those receiving saline, indicative of fibrotic development (Supplementary Fig. 5B). Picrosirius red staining show enhanced collagen deposition and increased fibres density in bleomycin treated mice, observed by light and fluorescence microscopy (Supplementary Fig. 5C). At day 28, marked skin thickening was observed in bleomycin treated mice by staining with H&E, Masson’s trichrome and picrosirius red (Fig. 5B, C) on saline treated mice. This, however, was significantly prevented by BMS-470539 treatment.

### Intradermal administration of BMS-470539 does not induce skin fibrosis

**Fig. 6:**
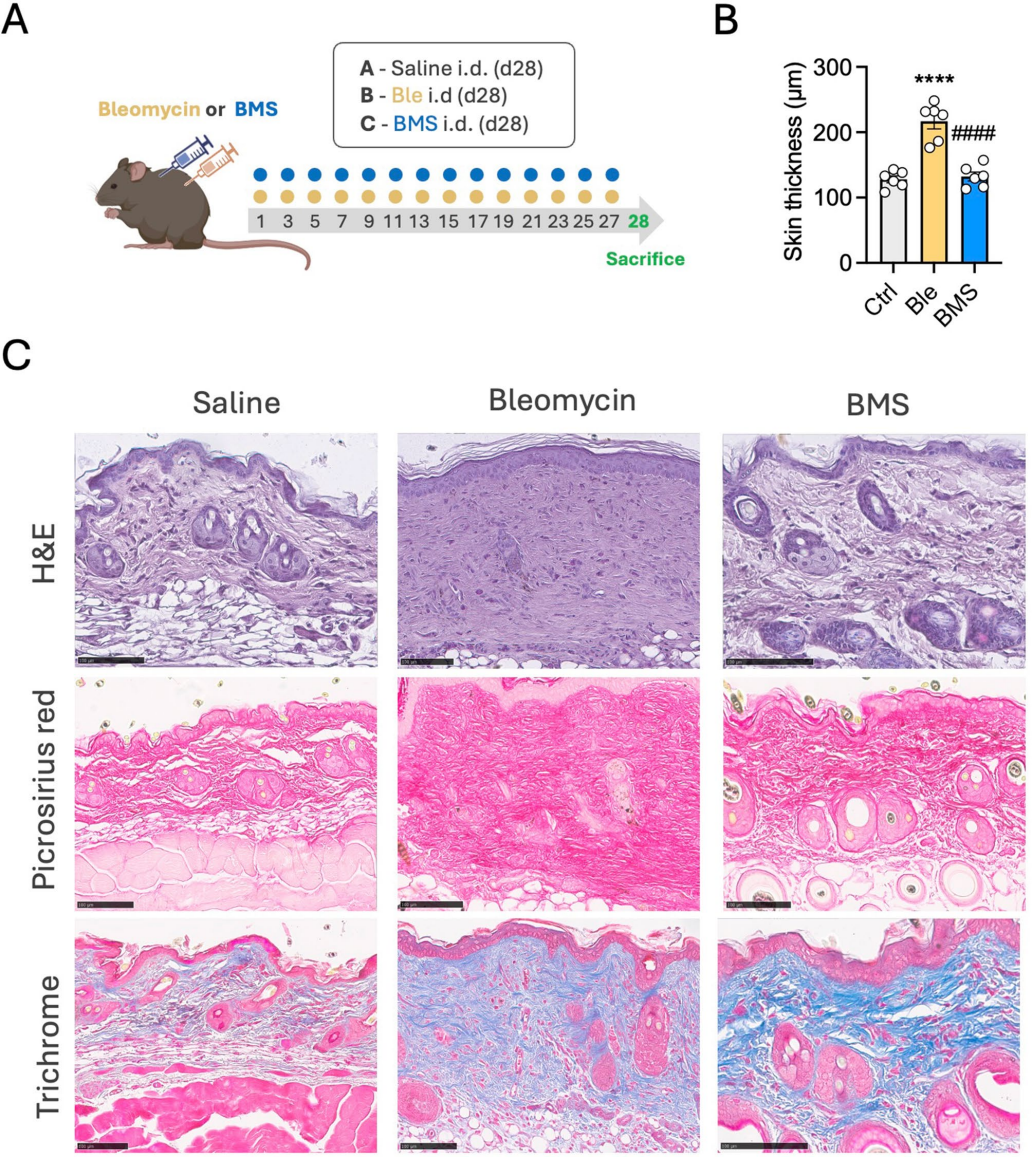
Absence of pro-fibrotic actions of BMS-470539 administered intradermally. (**A**) Diagram representing the experimental design used to test the potential pro-fibrotic actions of BMS-470539 when administered intradermally (i.d.). 50 µl of 1 mg/ml bleomycin or 3.6 mg/ml BMS-470539 were administered intradermally (i.d.) from day 1. (**B**) The dermal thickness of the H&E-stained sections was quantified in NDP.view2 in vehicle (Ctrl), bleomycin i.d. (Ble) or BMS-470539 i.d. (BMS) treated mice (**C**) Representative images of H&E, picrosirius red and Masson’s trichrome stained tissue sections of skin injected intradermally with saline, bleomycin or BMS-470539. Data represent the mean ± SEM of *n* = 6; one-way ANOVA, corrected with Tukey’s multiple comparisons test vs. Ctrl ( ****p < 0.0001) and vs. Ble (^####^*p* < 0.0001). Images were captured using a Nanozoomer slide scanner and scale bars represent 100 μm

**Fig. 7:**
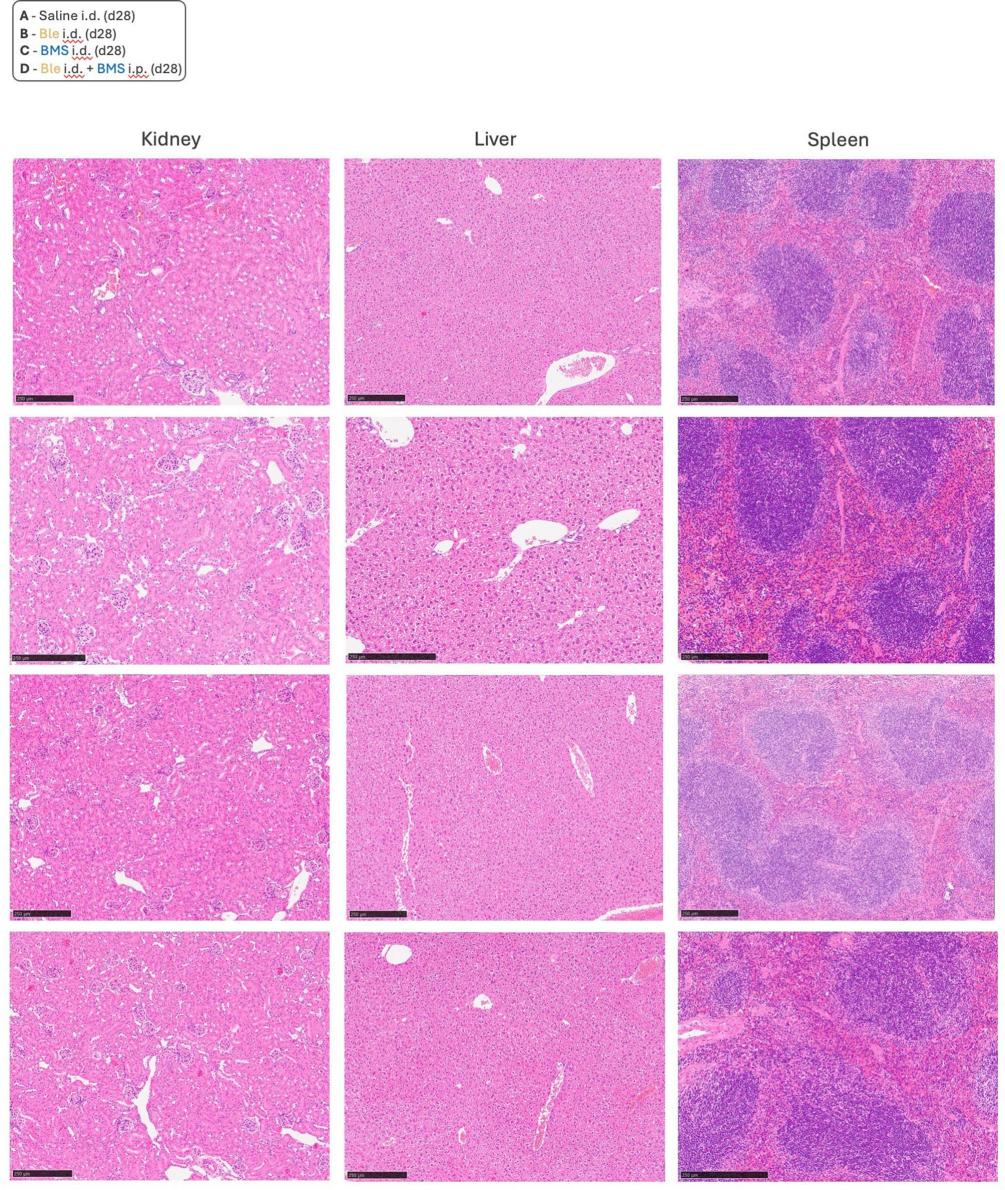
Absence of microscopic organ alteration in mice treated with BMS-470539. **A**) Tissue sections from the kidneys, livers and spleens were collected at day 28. Sections were stained with H&E to assess for changes in organ health after treatment with BMS-470539 administered either intradermally (i.d.) or intraperitoneally (i.p) as an assessment of the systemic effects of BMS-470539 administration. Representative images of *n* = 6. Images were captured at using a Nanozoomer slide scanner and scale bars represent 250 μm

Bleomycin exhibits a complex cytotoxic activity combining pro-apoptotic and pro-senescence effects together with generation of oxidative stress [21, 22]. Although direct evidence is scarce, it has typically been assumed that the pro-fibrotic action of bleomycin derives directly from its ability to induce cellular senescence, irrespective of other actions. It was then important to determine whether BMS-470539 administered intradermally, would be able to induce skin fibrosis. A group of mice received i.d. BMS-470539 using the same administration protocol as for bleomycin, i.e. 50 µl BMS-470539 solution distributed in the four corners of 1cm^2^ area of dorsal skin area, every other day for 27 days (Fig. 6A). No signs of skin thickening were observed on i.d. BMS-470539 treated mice nor cellular infiltrates (Fig. 6B, C) and this was clearly different from the bleomycin treated group. In addition, bleomycin treatment caused a trend for changes in the circulation including a reduction in red blood cells and haemoglobin, and an increase in circulating neutrophils (Supplementary Fig. 6). These systemic changes however were not present in i.d. BMS-470539 treated mice. Importantly, a histopathological screening of relevant organs did not reveal any apparent changes in morphology that could indicate toxicity with BMS-470539 administered neither intraperitoneally nor intradermally, as there is no precedent in literature of continuous administration of BMS-470539 over a period of 2 weeks (Fig. 7).

## Discussion

Despite human knowledge growing at an exponential rate driven by advances in science and technology, this is not resulting in an exponential increase in therapeutic solutions reaching patients. A total of 38 new drugs were approved by the FDA in 1999, while only 55 in 2023, a small improvement in a quarter of a century [23] besides the large increases in R&D spending. Fibrotic diseases are particularly affected by the scarcity of available disease-modifying treatments, despite intense efforts in the field [24]. To date, only two specific anti-fibrotic agents are approved, nintedanib (Ofev^®^) and pirfenidone (Esbriet^®^), both indicated for the treatment of idiopathic pulmonary fibrosis (IPF). Nintedanib, a tyrosine kinase inhibitor, is believed to act directly on the fibroblasts inhibiting their proliferation and migration and other pro-fibrotic functions [25]. Pirfenidone, by a poorly understood mechanism, acts by preventing TGFβ induced collagen production by fibroblasts, exerting anti-fibrotic, as well as anti-inflammatory and antioxidant effects. The success of these two drugs in improving survival of IPF patients highlights the importance of directly targeting the stromal cells. However, no specific anti-fibrotic medicines are available for other fibrotic conditions like systemic sclerosis. Various approaches targeting the fibroblast have been explored, including TGFβ ligand specific neutralising antibodies like fresolimumab [26], inhibition of myofibroblast transdifferentiation [27], induction of myofibroblast apoptosis [10], targeting Janus kinase signalling [28], or using mechano-therapeutics to target mechanical signalling and matrix stiffness [29].

Understanding context-dependent differences is important to fully exploit the therapeutic benefit of complex interventions. This is particularly relevant when proposing the process of cellular senescence as a therapeutic strategy, as growing evidence suggests that this may be beneficial or detrimental depending on the pathophysiological context [30]. While senescence in chondrocytes (i.e. parenchymal cells) in a condition strongly associated with ageing like osteoarthritis may result in detrimental effects [31], senescence in aggressive synovial fibroblasts present in rheumatoid arthritis seems beneficial [12, 32]. Senescence occurring in cells bearing oncogenic DNA damage is beneficial to prevent cancer [33]. However, if this process occurs in an organism with a deficient immune system unable to clear these cells off the tissues, it may result in additional tissue damage [34]. Then, a biological process which is present in all living organisms and highly conserved through evolution must be beneficial. It is an alteration of when, where, to what extent and how it is regulated, what leads to pathological consequences [30].

We previously reported that induction of a senescence-like phenotype on synovial cells improved arthritis. Here, using the same molecule, BMS-470539, we show the translational potential of this process in the context of skin fibrosis. We were able to recapitulate a similar phenotype in both healthy and scleroderma derived dermal fibroblasts, where the addition of this compound was associated with several markers of senescence like reduced proliferation, increased lysosomal accumulation and increased expression of SA-βGal and lipofuscin accumulation.

A distinctive and relevant distinctive feature of BMS-470539 senescence-like activity was the lack of pro-inflammatory SASP, a common characteristic of senescent cells. The release of a SASP by senescent cells is believed to play an important role in flagging the presence of these cells to induce their own clearance by the immune system [35, 36]. However, the drug reduced CCL2 release to a similar extent as the known anti-fibrotic compounds nintedanib and pirfenidone. Moreover, BMS-470539 secretome did not cause either the induction of secondary senescence, a process in which senescent cells induce further senescence in surrounding cells via paracrine or juxtacrine mechanisms [37].

Having characterised the unique features of these senescence-like actions on dermal fibroblasts, we then aimed at addressing the potential of this compound as an anti-fibrotic molecule. Using in vitro cultured cells, we show that BMS-470539 reduced the expression of ⍺SMA, slowed down the closure of the gap on a scratch assay and downregulated biological processes like angiogenesis, integrin signalling or cell migration among others, addressed using a whole genome transcriptomic approach. Specific genes include cell cycle regulators (*CDK1*,* CENPF*,* CDC27*), genes involved in myofibroblast contractility (*ACTA2*,* CNN2*,* TPM2*) or TGFβ function related genes (*TGFB1I1*,* GDF6*,* P4HA1*). In vivo, using the well-established bleomycin-induced skin fibrosis model, the drug, applied as a therapeutic schedule once fibrosis has been established (day 14), significantly reduced the development of fibrosis, observed by reduced skin thickness by ~ 50%. In this case BMS-470539 was administered intraperitoneally. Although this model is considered the gold-standard in the field for pre-clinical testing of new molecules, it lacks important aspects of the human condition [38]. While a direct chemical insult drives fibrosis in this mouse model, the presence of an autoimmune component and immune dysregulation are central for the development of human scleroderma.

In certain contexts, the presence or the accumulation of senescent cells has been suggested to be responsible, at least in part, for causing fibrosis [39, 40]. This is a controversial area though, as senescence cells have also been described as promoters of wound healing and tissue repair [41–44]. Besides this still unresolved matter (which may likely be explained by considering contextual aspects), we nonetheless aimed to address if the senescence-like actions of BMS-470539 could result into pro-fibrotic activity after repeated intradermal administrations, following the same procedure and schedule used for bleomycin. We demonstrate that fibrosis is completely absent in the skin of mice treated intradermally with BMS-470539, as well as at systemic level. In fact, previous studies have reported that fibrosis in the bleomycin model is caused by excessive Fas-mediated apoptosis and inflammatory response [45] rather than senescence. In a model of bleomycin-induced lung fibrosis, it has also been shown that clearance of senescent cells, although improving pulmonary function, it did not lead to reduction in lung fibrosis [39]. Nonetheless, intensive research on the targeting of senescent cells with senolytics and senomorphics is ongoing [46] and may be proven effective in certain cases. The time of treatment, however, may result crucial for this success, as simply by eliminating senescent cell in an advanced stage of fibrosis may not help restore the parenchymal and mesenchymal composition of a healthy tissue.

Of relevance from the therapeutic translational perspective, we did not observe any signs of toxicity on internal organs at microscopic level after prolonged administration of BMS-470539 for up to two weeks. We did not find either evidence of loss of activity on fibroblasts from patients carrying *MC1R* gene variants, another crucial aspect in drug development, as it has been estimated that > 30% of GPCR drug targets carry missense variants and their therapeutic use may benefit from pharmacogenetics approaches [47]. The only variant we identified in our previous study to interfere with BMS-470539 senescence-like activity, D294H, was not present in the fibroblasts available for this study, and hence this could not be addressed on this occasion. Importantly, other molecules targeting MC_1_ and other melanocortin receptors have demonstrated potential for fibrotic disorders [8, 48], including the small molecule MC_1_ selective agonist dersimelagon (MT-7117) [49]. Here we demonstrate that this molecule does not present senescence-like activity like BMS-470539 (MC_1_ selective) neither does the pan-agonist ⍺MSH. This may be suggesting that an additional mode of action or activity exclusively engaged by BMS-470539 may be operating to induce this senescence-like activity, and likely this may involve the engagement of a second target, independently of MC_1_. Further research will help to elucidate this unique mode of action.

In conclusion, we provide evidence that the senescence-like actions of the compound BMS-470539 could be beneficial in settings of fibrosis by preventing fibroblasts proliferation and activation without the toxicity derived from a pro-inflammatory SASP, as typical pro-senescence drugs do. Some aspects remain to be known, like for example, whether a combination with senolytic drugs will further enhance the in vivo efficacy of BMS-470539, how the anti-inflammatory actions derived directly from MC_1_ agonism contribute to the efficacy, or how the actions of BMS-470539 be affected in the context of fibroblast heterogeneity. Future studies will address these important questions and hopefully reveal the still elusive mechanism of action of BMS-470539. Finally, this work also remarks the importance of considering the fibroblast as a direct therapeutic target, and the translational impact of exploring shared pathogenic mechanisms across multiple diseases, as we demonstrate, in our case, from arthritis to fibrosis.

## Supplementary Information

Below is the link to the electronic supplementary material.

Supplementary Material 1

## Data Availability

RNA sequencing dataset has been deposited at Gene Expression Omnibus (GEO) Database (https://www.ncbi.nlm.nih.gov/geo/), with accession number GSE279858.

## Abbreviations

⍺MSH: ⍺-melanocyte stimulating hormone
FC: fold change
HV: healthy volunteer
i.d.: intradermal
i.p.: intraperitoneal
MC_1_: melanocortin 1 receptor
RA: rheumatoid arthritis
SA-βGal: senescence-associated β-galactosidase
SASP: senescence associated secretory phenotype
SSc: systemic sclerosis
