# Supplementary material for: The senescence-like activity of BMS-470539 is associated with anti-fibrotic actions in models of dermal fibrosis
Source: Arthritis Res Ther. 2025 Sep 30;27:187. doi: 10.1186/s13075-025-03635-3 (PMC12482462; doi:10.1186/s13075-025-03635-3)
Supplement: Supplementary file 1 — Supplementary Material 1 [file 13075_2025_3635_MOESM1_ESM.pdf]

Supplementary Figures and Tables

The senescence-like activity of BMS-470539 is associated with anti-fibrotic actions in models of dermal fibrosis

Camilla S.A. Davan-Wetton, Natalya Khodeneva, Christopher P Denton, David J Abraham, Mauro Perretti, Trinidad Montero-Melendez

Supplementary Figure 1

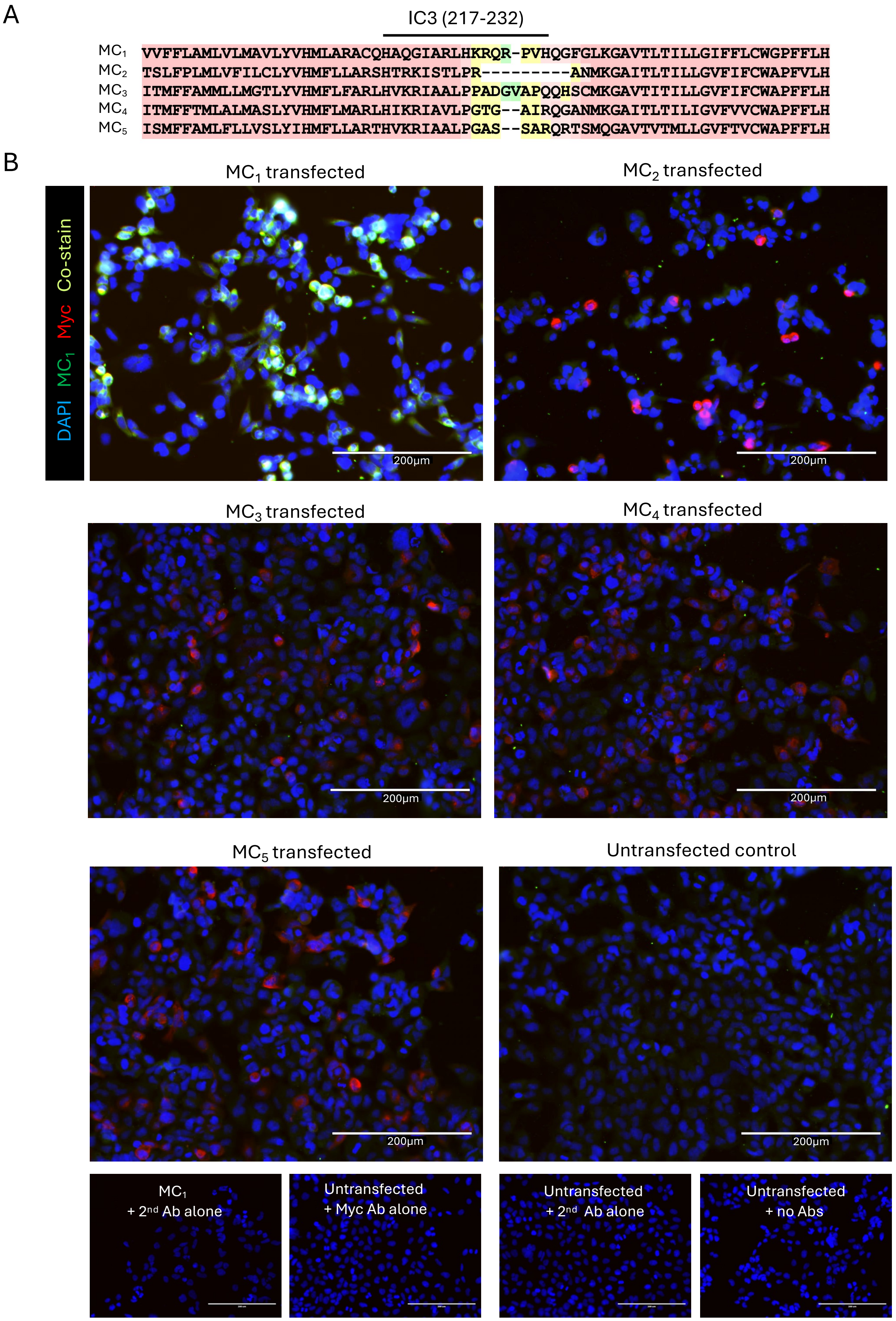

Supplementary Figure 1. ***In vitro*** **validation of an anti-MC_1 receptor antibody on transfected HEK293T cells**. **A)** Amino acid sequence alignment of the five human melanocortin receptors. The peptide used as immunogen to generate the anti-MC_1 receptor rabbit polyclonal antibody (Alomone, Cat. No. AMR-020) corresponds to the intracellular domain 3, a region of high variability. **B)** HEK293T cells were transiently transfected with each of the melanocortin receptors, using Myc-tagged expression vectors, and stained 24 h later with an anti-Myc antibody (Cell Signalling Technology) and the anti-MC_1 receptor rabbit polyclonal antibody (Alomone). Yellow colour indicates co-staining of MC_1 with the Myc tag. Images were captured at 20X and scale bars represent 200 μm.

Supplementary Figure 2

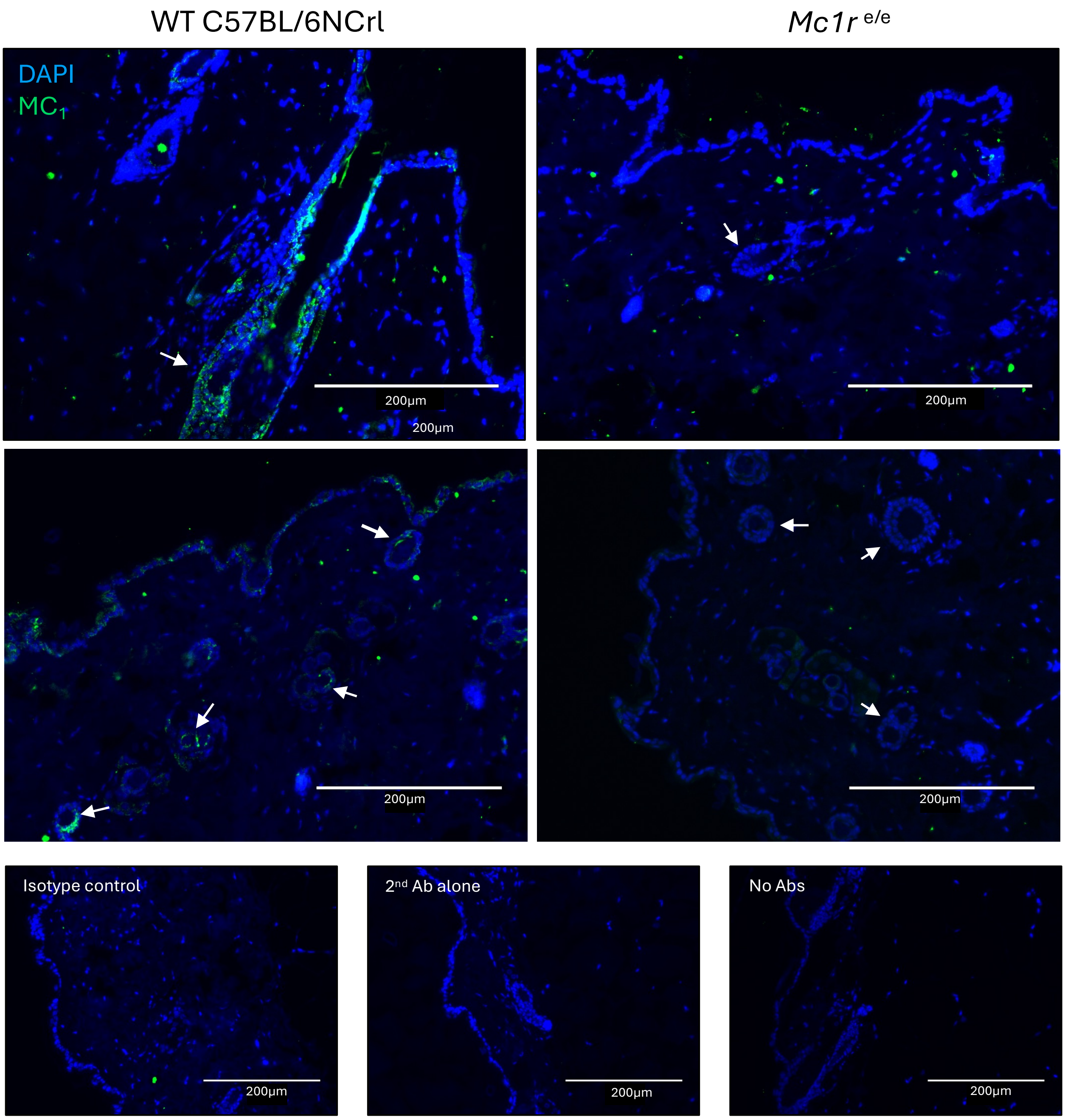

Supplementary Figure 2. *In vivo* validation of an anti-MC$_1$ receptor antibody using *Mc1r* $^{e/e}$ mouse tissues. Formalin-fixed paraffin-embedded skin tissues from wild type (WT) C57BL/6NCrl and *Mc1r* $^{e/e}$ mice (lacking a functional MC$_1$ receptor) were subjected to immunofluorescence staining using the anti-MC$_1$ receptor rabbit polyclonal antibody (Alomone, Cat. No. AMR-202). Images were captured at 20X and scale bars represent 200 µm. While arrows indicate hair follicles where MC$_1$ expression is expected.

Supplementary Figure 3

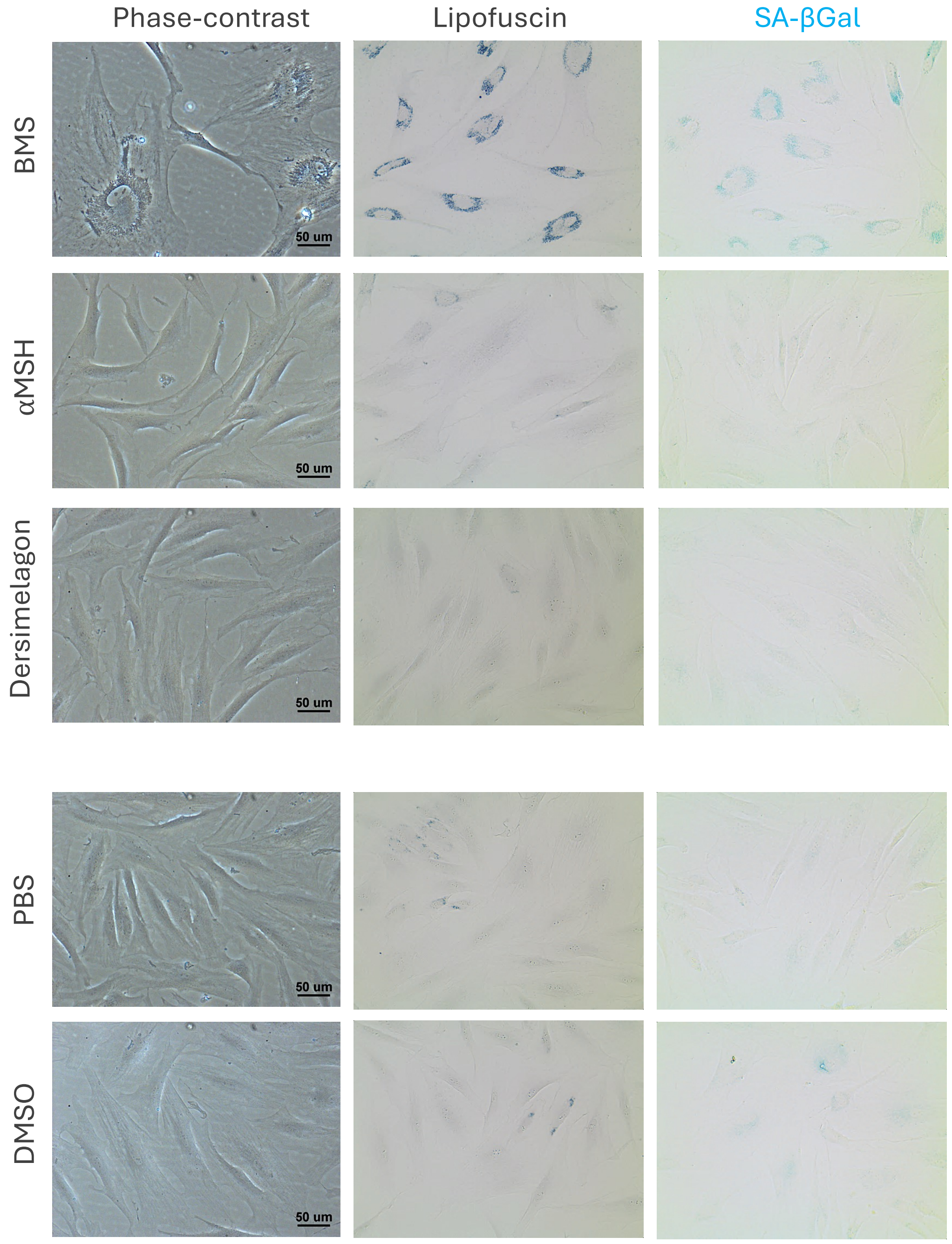

Supplementary Figure 3. Induction of senescence markers by BMS-470539, dersimelagon and αMSH on *in vitro* cultured dermal fibroblasts. Systemic sclerosis (SSc) fibroblasts were treated with vehicle (PBS or DMSO), 10 μM BMS-470539, 10 μM dersimelagon or 10 μM αMSH every other day for 6 days. Cells were stained for senescence associated β-galactosidase (SA-βGal) activity or lipofuscin using Sudan black B. Images were captured at 40X with a light microscope. Representative images are shown.

Supplementary Figure 4

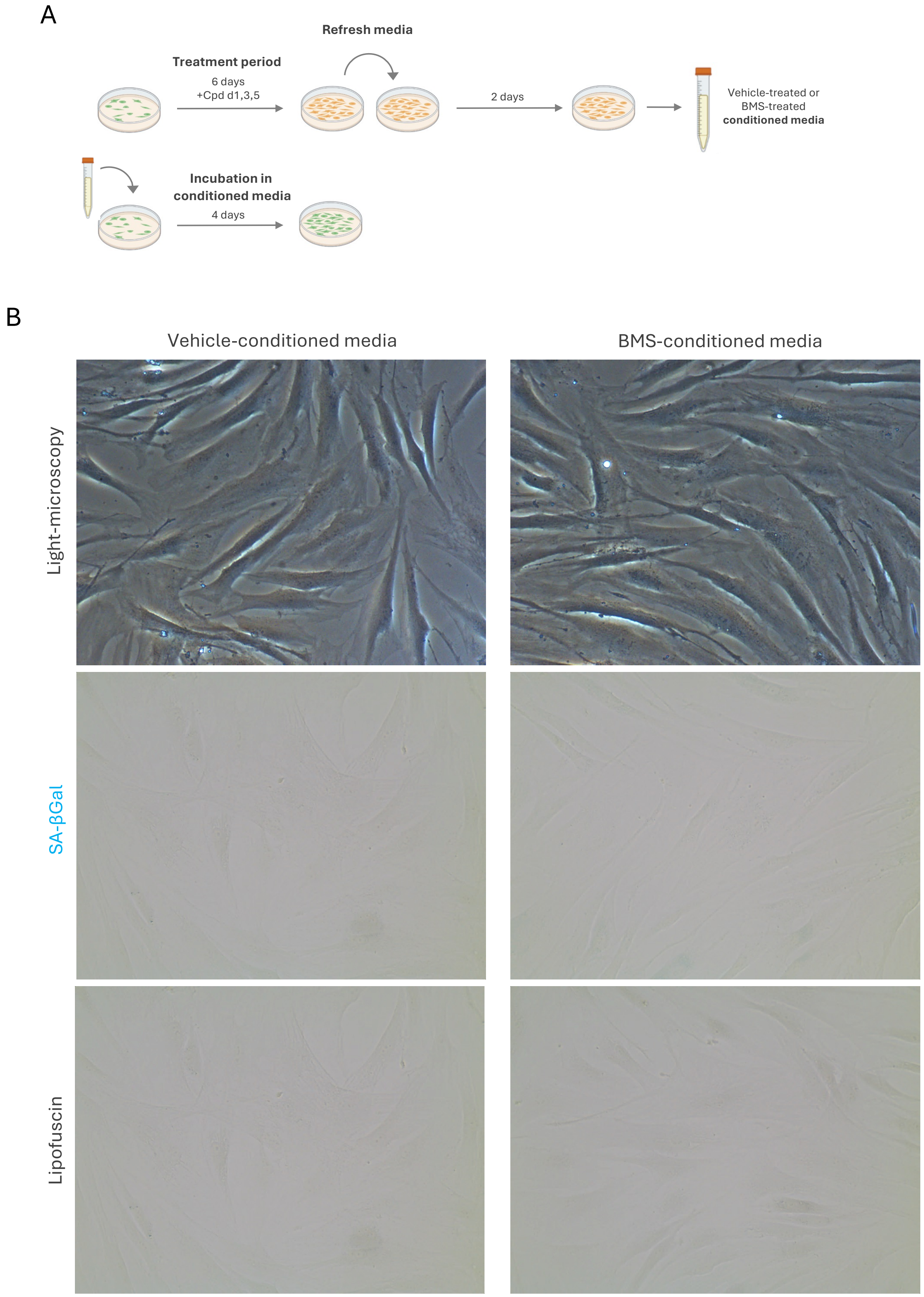

Supplementary Figure 4. Absence of secondary senescence on *in vitro* cultured human dermal fibroblasts treated with BMS-conditioned media. **A)** Diagram representing the procedure to obtain conditioned media. Systemic sclerosis (SSc) fibroblasts were treated with vehicle (Ctrl) or 10 μM BMS-470539, every other day for 6 days. Cells were washed to remove any remining BMS-470539 compound and fresh media added. Cells were incubated for 2 days to allow the release of mediators. Media was then collected, filtered and used on newly plated cells for 4 days. **B)** Cells were analysed by visualising the presence of a perinuclear lysosomal ring (light microscopy images), senescence associated β-galactosidase (SA-βGal) activity and by lipofuscin staining using Sudan black B. Images were captured at 40X. Representative images of n=3.

Supplementary Figure 5

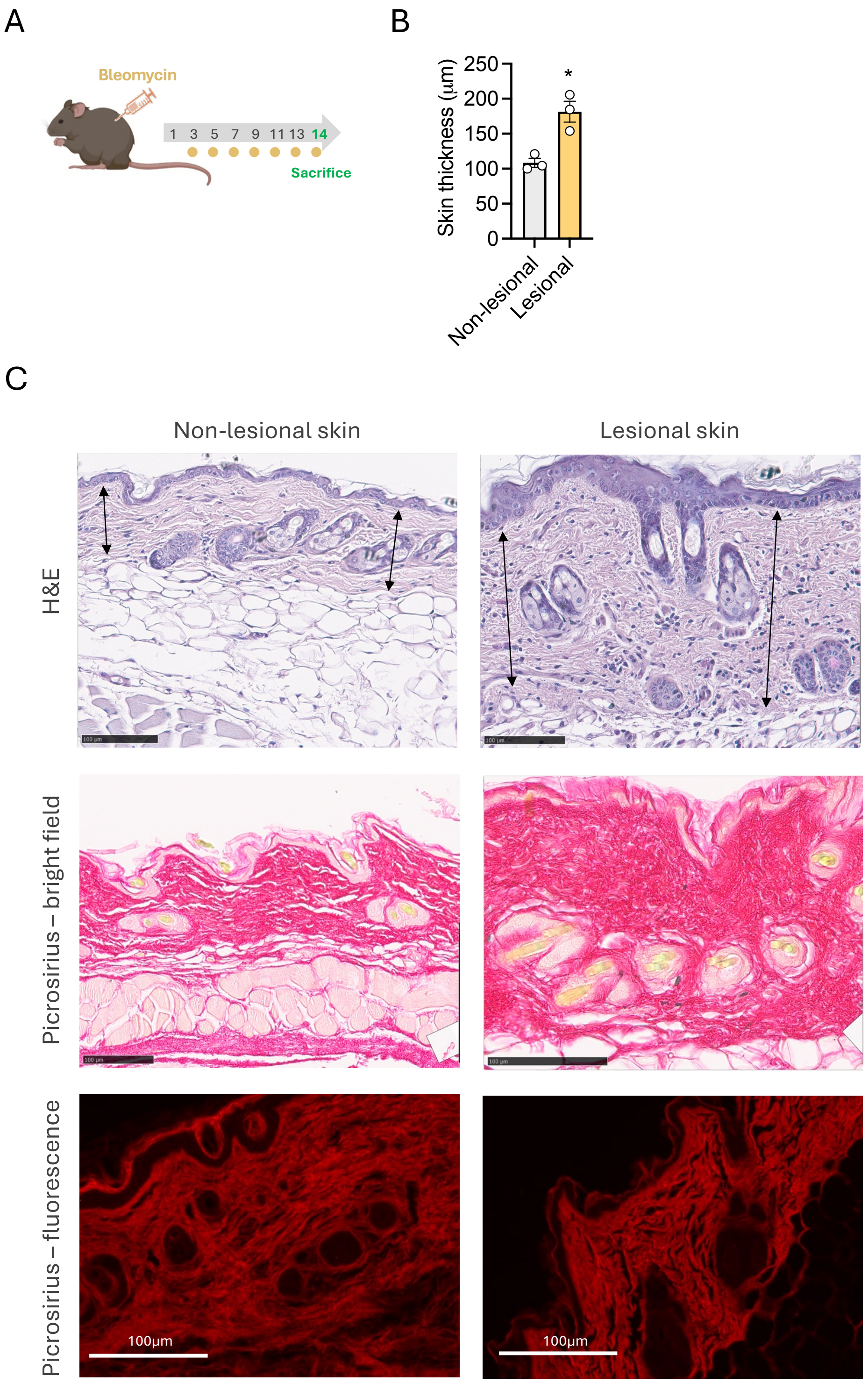

Supplementary Figure 5. Establishment of fibrosis on the bleomycin-induced skin fibrosis murine model at day 14. **A)** Diagram representing the experimental design used to induce dermal fibrosis. 1 mg/ml bleomycin was administered intradermally (i.d.) every other day from day 1 until day 14. **B)** The dermal thickness of the H&E-stained sections was quantified in NDP.view2. **C)** Representative images of H&E and picrosirius red stained tissue sections of skin from saline and bleomycin treated mice as a visualisation of skin thickness and collagen deposition Picrosirius red sections were visualised by bright field and fluorescence microscopy. Data represent the mean ± SEM ($n=3$; unpaired t-test, $^*p<0.05$). Images were captured at using a Nanozoomer slide scanner and scale bars represent 100 μm.

Supplementary Figure 6

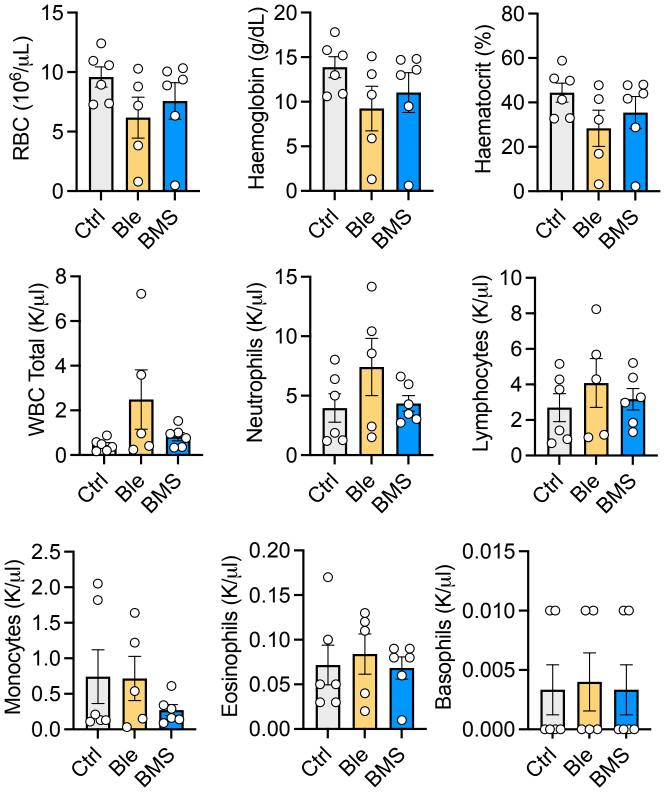

Supplementary Figure 6. Systemic changes in mice treated intradermally with bleomycin or BMS-470539. 50µl of saline, 1 mg/ml bleomycin or 3.6 mg/ml BMS-470539 were administered intradermally (i.d.) from day 1 until day 27. Mice were sacrificed at day 28 and blood collected by cardiac puncture. Blood was analysed using a ProCyte Dx Haematology Analyzer (IDEXX). Data represent mean ± SEM of n=4 and was analysed by paired one-way ANOVA *vs.* control followed by Dunnett's multiple comparisons test.

Supplementary Table 1

Donor	Sex	Age (years)	Diffuse or limited	Auto-antibodies	Co-morbidities	Medication	*MC1R* genotype
**HV101**	F	54	n/a	n/a	n/a	n/a	R151C
**HV102**	M	73	n/a	n/a	n/a	n/a	WT
**HV103**	F	54	n/a	n/a	n/a	n/a	R151C
**HV104**	F	73	n/a	n/a	n/a	n/a	WT
**SSc1**	F	53	Diffuse	ARA	ILD, OA, aortic valve replacement	Mycophenolate	V60L , R160W
**SSc5**	F	42	Diffuse	-	Lung fibrosis, severe midgut disease	Lansoprazole, Proventil	R151C
**SSc7**	F	47	Diffuse	Anti-Scl-70	Hypothyroidism, thalassaemia trait	Iloprost, Mycophenolate mofetil	WT
**SSc8**	F	41	Diffuse	Anti-Scl-70	ILD, myocardial involvement, inflammatory arthritis	No active medications	V60L , R151C
**SSc10**	F	53	Diffuse	ANA, ENA, anti-Ro, anti-Scl-70	Myositis overlap, cardiac involvement, ILD, RP, hypothyroidism, AF	Hydroxychloroquine, Iloprost, Prednisolone, Rituximab	V60L , R151C
**SSc11**	F	52	Diffuse	-	None	Lansoprazole	WT
**SSc12**	F	45	Limited	ARA	Carcinoid of appendix, hypothyroidism, RP	Folic acid, Levothyroxine, Methotrexate, Sildenafil	WT
**SSc13**	M	51	Diffuse	Anti-RNA pol III	Inflammatory arthritis, GAVE, anaemia, ischaemic heart disease, depression	Tocilizumab	V60L , R151C
**SSc14**	F	51	Diffuse	-	Hypomagnesemia, hypothyroidism, ILD, anaemia, osteoporosis	Iloprost, Prednisolone, Myfortic, Adizem-SR	V92M , R163Q , T314T
**SSc15**	F	44	Limited	Anti-Scl-70	Inflammatory arthropathy, digital ulcer, myocardial involvement	Iloprost, Hydroxychloroquine, Sildenafil	V60L , R151C

Supplementary Table 1. Characteristics of healthy volunteers (HV), and systemic sclerosis patients (SSc) which donated skin samples for the obtention of dermal fibroblasts. AF: atrial fibrillation; ANA: anti-nuclear antibody; Anti-RNA poly III: anti-RNA polymerase III antibody; Anti-Ro: anti-Sjogren's-syndrome-related antigen A antibody; Anti-Scl-70: anti-topoisomerase 1 antibody; ARA: anti-reticulin antibody; ENA; anti-extractable nuclear antigen antibody; GAVE: gastric antral vascular ectasia; ILD: interstitial lung disease; OA: osteoarthritis; RP: Raynaud's phenomenon. *MC1R* gene variants with strong association with red hair are highlighted in red.

Supplementary Table 2

Haplotypes	No.	%	RHC phenotype
**WT**	3	30	n/a
**V60L / R151C**	4	40	r / R
**V60L / R160W**	1	10	r / R
**V92M / R163Q**	1	10	r / r
**R151C**	1	10	R

Supplementary Table 2. Summary of *MC1R* genotyping results for the systemic sclerosis (SSc) patients fibroblasts (n=10) used in this study indicating the number and proportion of donors carrying the specified haplotype and the red hair colour (RHC) phenotype as described in literature (R= high association with red hair (in red), r= weak association with red hair).

Supplementary Table 3

Variant	Substitution	RHC	AA	Aa	aa	p	q	%
**F45L**	133 T>C	r	96	1	0	0.995	0.005	1.0
**V60L**	178 G>T	r	64	32	1	0.825	0.175	34.0
**D84E**	252 C>A	R	96	1	0	0.995	0.005	1.0
**V92M**	274 G>A	r	80	14	3	0.897	0.103	17.5
**T95M**	284 C > T	R	96	1	0	0.995	0.005	1.0
**R151C**	451 C>T	R	85	12	0	0.938	0.062	12.4
**R160W**	478 C>T	R	80	17	0	0.912	0.088	17.5
**R163Q**	488 G>A	r	88	8	1	0.948	0.052	9.3
**V193L**	577 G>A	R	96	1	0	0.995	0.005	1.0
**F196L**	586 T>C	R	96	1	0	0.995	0.005	1.0
**D294H**	880 G>C	R	94	2	1	0.979	0.021	3.1

Supplementary Table 3. Summary of *MC1R* genotyping results for the systemic sclerosis (SSc) patients DNA samples (n=97) used in this study indicating the individual variants identified, nucleotide position and substitution, red hair colour (RHC) phenotype (highly associated shown in red), number of patients homozygotes for mayor allele (AA), number heterozygotes (Aa), number of homozygotes for minor allele (aa), mayor allele frequency (p), minor allele frequency (q) and proportion of patients carrying that variant (%).

Supplementary Table 4

Haplotypes	No.	%	RHC
WT	18	18.6	n/a
F45L	1	1.0	r
V60L	21	21.6	r
V60L / V92M	3	3.1	r / r
V60L / R151C	4	4.1	r / R
V60L / R160W	1	1.0	r / R
V60L / R163Q	3	3.1	r / r
V60L / V193L	1	1.0	r / R
V60L / D294H	1	1.0	r / R
D84E	1	1.0	R
V92M	11	11.3	r
V92M / R160W	3	3.1	r / R
T95M	1	1.0	R
R151C	8	8.2	R
R160W	11	11.3	R
R160W / R163Q	1	1.0	R / r
R160W / D294H	1	1.0	R / R
R163Q	5	5.2	r
F196L	1	1.0	R
D294H	1	1.0	R

Supplementary Table 4. Summary of *MC1R* genotyping results for the systemic sclerosis (SSc) patients DNA samples (n=97) used in this study indicating the number and proportion of donors carrying the specified haplotype and the red hair colour (RHC) phenotype as described in literature (R= high association with red hair, in red, r= weak association with red hair).

Supplementary Table 5

Gene Symbol	Transcript ID	Gene Name	Fold change	p value
**ACTA2**	NM_001613	Actin alpha 2, smooth muscle	0.25	0.038
**BAG2**	NM_004282	BAG cochaperone 2	0.80	0.015
**BCL2L2**	NM_004050	BCL2 like 2	1.23	0.003
**BIRC5**	NM_001168	Baculoviral IAP repeat containing 5	0.53	0.007
**BOK**	NM_032515	BCL2 family apoptosis regulator BOK	0.79	0.027
**CALD1**	NM_033138	Caldesmon 1	0.56	0.010
**CAVIN2**	NM_004657	Caveolae associated protein 2	0.40	0.040
**CDC27**	NM_001256	Cell division cycle 27	0.78	0.022
**CDCA3**	NM_031299	Cell division cycle associated 3	0.54	0.039
**CDK1**	NM_001786	Cyclin dependent kinase 1	0.59	0.024
**CDKN1A**	NM_078467	Cyclin dependent kinase inhibitor 1A	1.91	0.043
**CDKN3**	NM_005192	Cyclin dependent kinase inhibitor 3	0.53	0.003
**CEMIP**	NM_018689	Cell migration inducing hyaluronidase 1	0.80	0.013
**CENPF**	NM_016343	Centromere protein F	0.59	0.033
**CENPH**	NM_022909	Centromere protein H	0.67	0.026
**CNN1**	NM_001299	Calponin 1	0.35	0.004
**CNN2**	NM_004368	Calponin 2	0.66	0.014
**COL5A2**	NM_000393	Collagen type V alpha 2 chain	0.82	0.049
**EIF2S2**	NM_001316364	Eukaryotic translation initiation factor 2 subunit beta	0.75	0.024
**FHL1**	NM_001159699	Four and a half LIM domains 1	0.35	0.041
**FSTL1**	NM_007085	Follistatin like 1	0.82	0.023
**GDF6**	NM_001001557	Growth differentiation factor 6	0.38	0.034
**GLB1**	NM_000404	Galactosidase beta 1	1.39	0.032
**GTSE1**	NM_016426	G2 and S-phase expressed 1	0.55	0.026
**IFI6**	NM_002038	Interferon alpha inducible protein 6	3.31	0.006
**IL7R**	NR_120485	Interleukin 7 receptor	0.54	0.037
**INSIG1**	NM_001346592	Insulin induced gene 1	4.37	0.008
**ITGA1**	NM_181501	Integrin subunit alpha 1	0.82	0.026
**ITGA3**	NM_002204	Integrin subunit alpha 3	0.83	0.042
**LAMP1**	NM_005561	Lysosomal associated membrane protein 1	1.49	0.001
**LDLR**	NM_001195799	Low density lipoprotein receptor	2.71	0.021
**LMOD1**	NM_012134	Leiomodin 1	0.38	0.012
**LOXL1**	NM_005576	Lysyl oxidase like 1	0.71	0.020
**MIR503HG**	NR_024607	MIR503 host gene	0.29	0.033
**MKI67**	NM_001145966	Marker of proliferation Ki-67	0.53	0.019
**MMP14**	NM_004995	Matrix metallopeptidase 14	1.48	0.006
**MMP2**	NM_001127891	Matrix metallopeptidase 2	1.75	0.010
**MVK**	NM_000431	Mevalonate kinase	2.26	0.002
**MYL6**	NM_079423	Myosin light chain 6	0.66	0.039
**MYO1B**	NM_001161819	Myosin IB	0.60	0.030
**NGF**	NM_002506	Nerve growth factor	0.52	0.032
**P4HA1**	NM_001142595	Prolyl 4-hydroxylase subunit alpha 1	0.83	0.017
**S100A16**	NM_001317007	S100 calcium binding protein A16	0.52	0.042
**SERPINE1**	NM_000602	Serpin family E member 1	0.47	0.018
**TAGLN**	NM_003186	Transgelin	0.55	0.003
**TGFB1I1**	NM_001042454	Transforming growth factor beta 1 induced transcript 1	0.71	0.005
**TPM1**	NM_001018007	Tropomyosin 1	0.78	0.035
**TPM2**	NM_213674	Tropomyosin 2	0.59	0.009
**TRIAP1**	NM_016399	TP53 regulated inhibitor of apoptosis 1	1.20	0.032
**VEGFA**	NM_001287044	Vascular endothelial growth factor A	0.79	0.022

Supplementary Table 5. Details of selected genes provided in Figure 5E, related to RNA sequencing analysis (n=3). Fold change is calculated as the ratio of BMS-470539 treated cells *vs.* controls.
